# Accumulation of systematic TPM1 mediates inflammation and neuronal remodeling by phosphorylating PKA and regulating the FABP5/NF‐κB signaling pathway in the retina of aged mice

**DOI:** 10.1111/acel.13566

**Published:** 2022-02-11

**Authors:** Rong Li, Yuxiang Liang, Bin Lin

**Affiliations:** ^1^ School of Optometry The Hong Kong Polytechnic University Kowloon Hong Kong; ^2^ The State Key Laboratory of Brain and Cognitive Sciences The University of Hong Kong Pok Fu Lam Hong Kong

**Keywords:** aging, dendritic sprouting, inflammation, parabiosis, retina, tropomyosin 1

## Abstract

The molecular mechanisms underlying functional decline during normal brain aging are poorly understood. Here, we identified the actin‐associated protein tropomyosin 1 (TPM1) as a new systemic pro‐aging factor associated with function deficits in normal aging retinas. Heterochronic parabiosis and blood plasma treatment confirmed that systemic factors regulated age‐related inflammatory responses and the ectopic dendritic sprouting of rod bipolar (RBC) and horizontal (HC) cells in the aging retina. Proteomic analysis revealed that TPM1 was a potential systemic molecule underlying structural and functional deficits in the aging retina. Recombinant TPM1 protein administration accelerated the activation of glial cells, the dendritic sprouting of RBCs and HCs and functional decline in the retina of young mice, whereas anti‐TPM1 neutralizing antibody treatment ameliorated age‐related structural and function changes in the retina of aged mice. Old mouse plasma (OMP) induced glial cell activation and the dendritic outgrowth of RBCs and HCs in young mice, and yet TMP1‐depleted OMP failed to reproduce the similar effect in young mice. These results confirmed that TPM1 was a systemic pro‐aging factor. Moreover, we demonstrated that systematic TPM1 was an immune‐related molecule, which elicited endogenous TPM1 expression and inflammation by phosphorylating PKA and regulating FABP5/NF‐κB signaling pathway in normal aging retinas. Interestingly, we observed TPM1 upregulation and the ectopic dendritic sprouting of RBCs and HCs in young mouse models of Alzheimer's disease, indicating a potential role of TPM1 in age‐related neurodegenerative diseases. Our data indicate that TPM1 could be targeted for combating the aging process.

## Graphical abstract

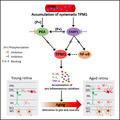

## 1 INTRODUCTION

Normal aging is associated with a progressive decline in brain function and increases vulnerability to neurodegenerative diseases in humans (Bishop et al., 2010; Mattson & Magnus, 2006). Considering that the life expectancy of the human population is increasing worldwide, understanding the mechanisms that are responsible for these age‐related alterations in the aging brain and finding therapeutic agents that might slow aspects of brain aging become increasingly critical. However, the basic molecular mechanisms of the aging process in the brain remain poorly understood.

Age‐related changes in systemic factors have been reported to drive aging phenotypes and impair cognition in the aging brain (Smith et al., 2015; Wyss‐Coray, 2016; Zhang et al., 2019). The discovery of such circulating factors will help develop new therapeutic agents to block the effects of pro‐aging factors and improve brain function in aged individuals. However, the factors mediating pro‐aging effects in old blood remain largely elusive. In the visual system, substantial age‐related alterations are reported in the aging retina (Liets et al., 2006; Samuel et al., 2011, 2014). As the retina is known as an extension of the brain (London et al., 2013), we used the retina as a window for exploring the effects of circulating pro‐aging factors on the brain. The dendrites of rod bipolar and horizontal cells in the aging retina are reported to sprout far beyond their normal strata within the outer plexiform layer into the photoreceptor nuclear layer (Liets et al., 2006; Samuel et al., 2014). Meanwhile, glial cells, including microglia and astrocytes, are found to be activated in the aging retina (Chen et al., 2019; Mansour et al., 2008; Ramírez et al., 2001; Telegina et al., 2018). Therefore, the changes in the dendritic sprouting of the retinal neurons and in the glial cell activation were used as structural biomarkers to determine potential pro‐aging factors. We performed heterochronic parabiosis, which surgically connects the circulatory systems of two organisms of different ages allowing sharing of the blood circulation and proteomics and identified the actin‐associated protein tropomyosin 1 (TPM1) as a new systemic pro‐aging factor underlying age‐related functional decline in the aging retina. Moreover, we demonstrated that systemic TPM1 accumulation elicited endogenous TPM1 upregulation in the aged retina via the phosphorylation of PKA and the activation of FABP5/NF‐κB signaling pathway. Interestingly, TPM1 elevation and the dendritic outgrowth of rod bipolar and horizontal cells, which were observed in normal aging retina, also occurred in young mouse models of Alzheimer's disease, indicating that TPM1 might play a similar role in age‐related neurodegenerative diseases as in normal brain aging. Together, our data suggest that TPM1 may be therapeutically targeted for combating the effects of aging in old age.

## 2 RESULTS

### 2.1 Heterochronic parabiosis induces age‐related inflammation and dendritic sprouting of rod bipolar cells and horizontal cells in young mice

The dendrites of rod bipolar (RBCs) and horizontal (HCs) cells were found to extend from their normal strata within the outer plexiform layer (OPL) to the outer nuclear layer (ONL) in the aging retina (Figure S1A–C), which is consistent with previous reports in aged retinas (Liets et al., 2006; Samuel et al., 2014). In young retinas, microglia were exclusively localized in the outer and inner plexiform layers (OPL and IPL) and ganglion cell layer (GCL) and were negative for CD68, a marker of activated microglia. With aging, microglia became activated and were positive for CD68 (Figure S1D–G). Some of the dendritic processes of activated microglia extended far beyond their normal confines of the OPL into the ONL (Figure S1D). Meanwhile, the numbers of microglia (Iba1^+^) and of activated microglia (CD68^+^Iba1^+^) were increased (Figure S1E–G), which were confirmed by western blotting analysis (Figure S1H–J). In young mice, astrocytes were entirely restricted to the NFL (Figure S1K). In aged retinas, astrocytes and Müller cells became activated with increased GFAP immunoreactivity (Figure S1K–M). The processes of reactive astrocytes became thicker, and many of thickened astrocytic processes extended into the IPL from their original tiled NFL. Electroretinography (ERG) recording showed markedly reduced amplitudes of dark‐ or light‐adapted a‐ and b‐waves in aged mice (Figure S1N–O), suggesting functional decline in the aging retina.

**FIGURE 1:**
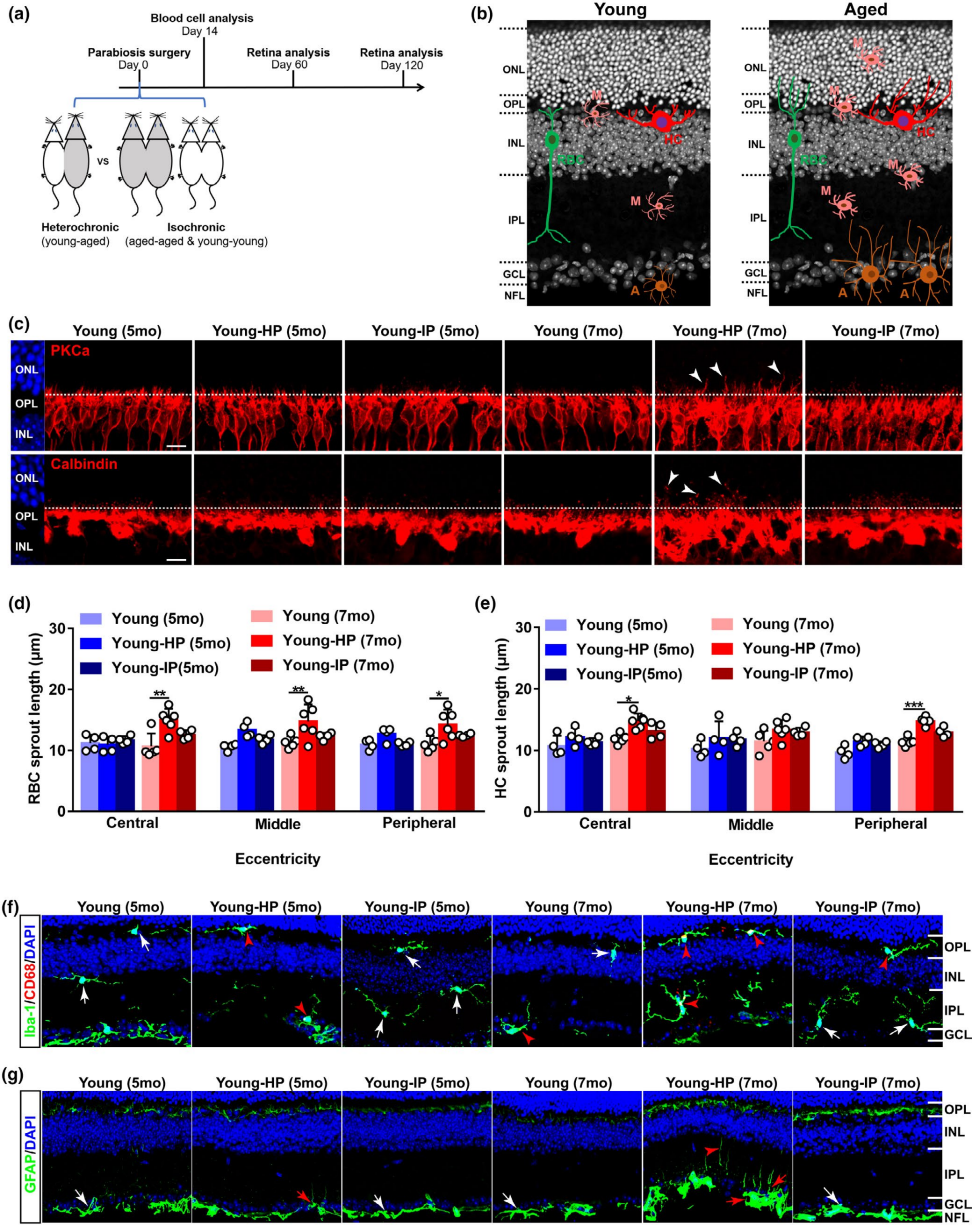
Heterochronic parabiosis induces age‐related alterations in neurons and glial cells in the young retina. (a) Schematic representation of the experimental strategy. Parabiosis was performed on young (3 months) and aged (11 months) C57BL/6J mice: heterochronic parabiosis (young‐aged) and isochronic parabiosis (young‐young & aged‐aged). Retinas were collected for analysis 60 and 120 days after surgery. (b) Schematic depicting age‐related alterations in neurons and glial cells in the retina. RBC: rod bipolar cell; HC: horizontal cell; M: microglial cell; A: astrocyte. (c) Representative confocal images of retinal sections from young mice after parabiosis stained with PKCα, a marker for RBCs (top row), and calbindin, a marker for HCs (bottom row). Arrowheads indicate the aberrant dendritic sprouting of RBCs and HCs to the ONL. The dotted line indicates the OPL/ONL border. Scale bars, 10 µm. Young‐IP, young isochronic parabionts; young‐HP, young heterochronic parabionts. (d–e) Quantification of the aberrant dendritic length of RBCs and HCs in young retinas after parabiosis. Three retinal sampling areas in retinal sections at 100 µm (central), 1 mm (middle), and 1.8 mm (peripheral) from the optic nerve head were captured, and the length between the tips of dendrites and the soma was measured. Data are presented as mean ± SEM and analyzed by one‐way ANOVA with Tukey's multiple comparison test, *n* = 4, 4, 4 mice in young (5 months), young‐HP (5 months), young‐IP (5 months), *n* = 5, 6, 4 mice in young (7 months), young‐HP (7 months), young‐IP (7 months). (f) Retina sections from young mice after parabiosis were stained with Iba‐1, a microglial cell marker, and CD68, a marker for activated microglia. Red arrowheads indicate CD68‐positive microglia, and white arrows show CD68‐negative microglia. Scale bars, 20 µm. (g) Retina sections from young mice after parabiosis were stained with GFAP, a marker for astrocytes and activated Müller cells in the retina. Activated astrocytes increase GFAP immunoreactivity and show clear signs of hypertrophy. The processes of reactive astrocytes become thicker, and many thickened astrocytic processes grow into the IPL from their original tiled NFL (red arrows). Red arrowhead indicates GFAP stained finer processes of activated Müller cells, and white arrows show the resting astrocytes. Scale bar, 20 µm. ONL, outer nuclear layer; OPL, outer plexiform layer; INL, inner nuclear layer; IPL, inner plexiform layer; GCL, ganglion cell layer; NFL, nerve fiber layer

Circulating factors in blood have been reported to regulate brain function during normal aging (Castellano et al., 2015; Katsimpardi et al., 2014; Ozek et al., 2018). To investigate whether systemic factors affected retina aging, we performed parabiosis, which surgically connected the circulatory systems of a WT mouse (C57BL/6J, white) with a Cx3cr1^+/GFP^ mouse (GFP mice, green) (Figure S2A). Two weeks after parabiosis, flow cytometric analysis showed that approximately 38% of blood cells was GFP positive in WT parabionts (Figure S2B–C), demonstrating the successful creation of a shared circulatory system after surgery. After that, we generated heterochronic pairs between 11‐month‐old (aged‐HP) and 3‐month‐old (young‐HP) mice as well as control groups of age‐matched pairs including isochronic aged (aged‐IP) and isochronic young (young‐IP) pairs (Figure 1a). The changes in the dendritic sprouting of RBCs and HCs and in the activation of glial cells including microglia and astrocytes between young and aged retinas were used as structural biomarkers to assay the effects of circulating factors following parabiosis (Figure 1b). We found that heterochronic parabiosis elicited age‐related dendritic sprouting of RBCs and HCs in young heterochronic parabionts 4 months after surgery (Figure 1c–e). Microglia became activated and astrocytes and Müller cells increased GFAP immunoreactivity, and some of astrocytic processes grew into the IPL from the NFL (Figure 1f–g) in young heterochronic parabionts 2 and 4 months after surgery. On the other side, heterochronic parabiosis attenuated age‐related dendritic sprouting of RBCs and HCs (Figure S2D–F) and suppressed glial activation in aged heterochronic parabionts. Microglia showed no typical dendritic extension to the ONL (Figure S2G), and astrocytes displayed less astrocytic processes to the IPL (Figure S2H) in aged heterochronic parabionts. Our results suggest that certain systemic factors in circulation regulate age‐related inflammation and neuronal remodeling in the aging retina.

### 2.2 Old plasma injection induces age‐related dendritic sprouting and visual function decline in young mice

**FIGURE 2:**
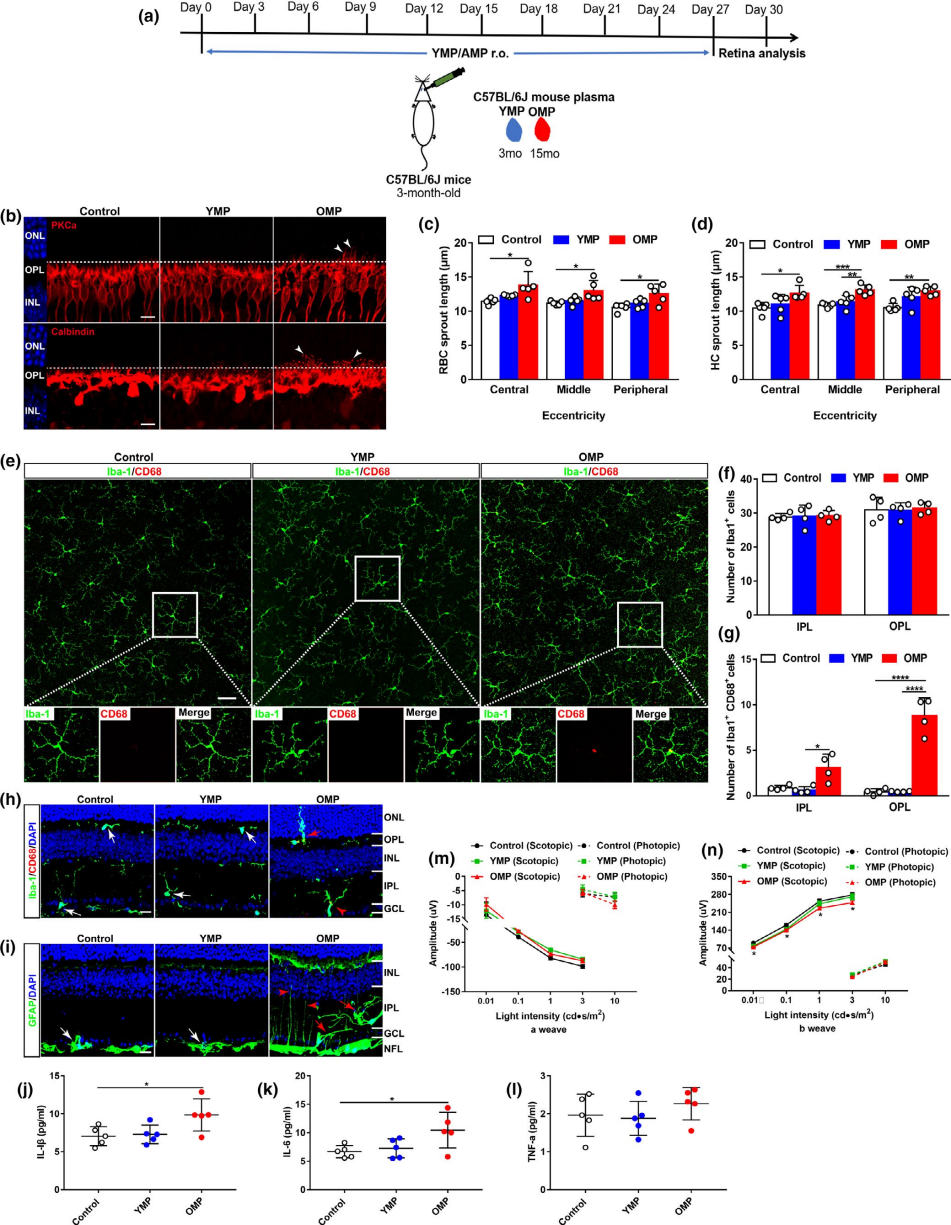
Old plasma injection induces age‐related dendritic sprouting, inflammation, and functional decline in young mouse retinas. (a) Schematic representation of the experimental design. Young mouse plasma (YMP) from young C57BL/6J mice (3 months) or old mouse plasma (OMP) from aged C57BL/6J mice (15 months) were retro‐orbitally injected into young C57BL/6J mice (3 months) for a total of ten times, 3 days apart, and retinas were collected for further studies at day 30. Mice in control groups received the same volume of phosphate‐buffered saline (PBS; pH 7.4) at the same time points. (b) Confocal images of RBCs (top row) and HCs (bottom row) in retinal sections from young mice after YMP or OMP treatment. Arrowheads indicate the aberrant dendritic sprouting of RBCs and HCs. Scale bars, 10 µm. (c–d) Quantification of the aberrant dendritic length of RBCs and HCs in young retinas after YMP or OMP treatment. Data were presented as mean ± SEM and analyzed by one‐way ANOVA with Tukey's multiple comparison test, *n* = 5 mice in each group. (e) Retina wholemounts from young mice after YMP or OMP treatment were stained with Iba‐1 and CD68. Confocal images focus on the OPL. The boxed regions are highly magnified at the bottom showing the colocalization of CD68 with microglia. Scale bars, 50 µm. (f–g) Quantification of the numbers of Iba‐1^+^ and of CD68^+^Iba‐1^+^ microglia in the IPL and OPL of whole‐mounted young retina after YMP or OMP treatment. Data are presented as mean ± SEM, *n* = 4 mice in each group. One‐way ANOVA analysis with Tukey's multiple comparison test. (h) Retina sections from young mice after YMP or OMP treatment were stained with Iba‐1 and CD68. White arrows indicate CD68‐negative microglia. Red arrowhead indicates CD68‐positive microglia, and red arrow indicates the dendritic extension of activated microglia from their original titled OPL to the ONL. Scale bars, 20 µm. (i) Retina sections stained with GFAP antibody. White arrows show the resting astrocytes. Red arrows show activated astrocytes, and red arrowheads indicate activated Müller cells. Scale bars, 20 µm. (j–l) ELISA analysis of pro‐inflammatory cytokines IL‐1β, IL‐6, and TNF‐α in young retinas after YMP and OMP treatment. Data are presented as mean ± SEM (*n* = 5 mice in each group) and analyzed by one‐way ANOVA with Tukey's multiple comparison test. (m–n) ERG recordings on young mice after YMP or OMP treatment. Data are presented as mean ± SEM (*n* = 12 mice in each group) and analyzed by one‐way ANOVA with Tukey's multiple comparison test (control vs. OMP, ^*^p < 0.05)

To confirm whether systemic factors regulated age‐related inflammation and neuronal outgrowth observed in the retina of heterochronic parabionts, we performed retro‐orbital injections with mouse plasma on young mice (Figure 2a). After ten injections over 1 month, we found that old mouse plasma (OMP) significantly elicited the incidence of the dendritic sprouting of RBCs and HCs to the ONL from the OPL in young retinas (Figure 2b–d). Moreover, OMP treatment activated microglia (Figure 2e–g,h), which extended their dendrites into the ONL from the OPL in young retinas (Figure 2h). OMP treatment did not cause change in the number of microglia (Iba1^+^) but in the number of activated microglia (CD68^+^Iba1^+^) in the young retina (Figure 2e–g). Similarly, OMP treatment induced hypertrophic astrocytes and increased the incidence of the extension of astrocytic processes to the IPL from the NFL, and activated Müller cells in young mice (Figure 2i). As a result, IL‐1β and IL‐6 were upregulated in OMP‐treated young retinas (Figure 2j–l). ERG recording showed markedly reduced amplitudes of dark‐adapted b‐wave in OMP‐treated young mice (Figure 2m–n), suggesting the impairment of rod photoreceptor function after OMP treatment.

On the contrary, old mice treated with young mouse plasma (YMP) exhibited attenuated dendritic sprouting of RBCs and HCs to the ONL from the OPL (Figure S3A–D). Moreover, YMP treatment ameliorated microglia activation showing decreased numbers of CD68^+^ microglia (Figure S3E–G) and reduced the incidence of the dendritic extension of activated microglia to the ONL from the OPL in aged mice (Figure S3H). In addition, YMP treatment reduced activation of astrocytes and the incidence of astrocytic processes that extended to the IPL from the NFL and of Müller cells (Figure S3I). As a result, pro‐inflammatory cytokines were downregulated in YMP‐treated aged retinas (Figure S3J–L). ERG recording showed that YMP treatment enhanced retinal function showing by higher b‐wave amplitudes of scotopic ERG relative to age‐matched controls and OMP‐treated mice (Figure S3M–N). Together, these results confirm that systemic soluble factors in blood mediate retina aging.

To directly confirm the effect of systemic factors, we treated BV2 cells, a murine microglial cell line, with mouse serum isolated from young (YMS) or old (OMS) mice in vitro. OMS treatment induced microglia activation (Figure S4A) and increased the percentage of active microglia (CD45^+^CD11b^+^) compared with control and YMS‐treated cells (Figure S4B–C). As a result, IL‐1β, IL‐6, and TNF‐α were elevated in OMS‐treated BV2 cells or in LPS‐treated BV2 cells (Figure S4D–F). Interestingly, OMS treatment did not induce the upregulation of cyclooxygenase‐1 (COX‐1) and COX‐2, key enzymes in the conversion of arachidonic acid to prostaglandins and other lipid mediators, in BV2 cells (Figure S4G–H). Moreover, OMS treatment elicited early apoptosis (Annexin‐V^+^PI^−^) in BV2 cells compared with control or YMS‐treated cells (Figure S4I–K). These results confirm that soluble factors in OMS trigger inflammation and cell apoptosis.

### 2.3 TPM1 is identified as a putative target protein by LC‐MS/MS and WB analysis

**FIGURE 3:**
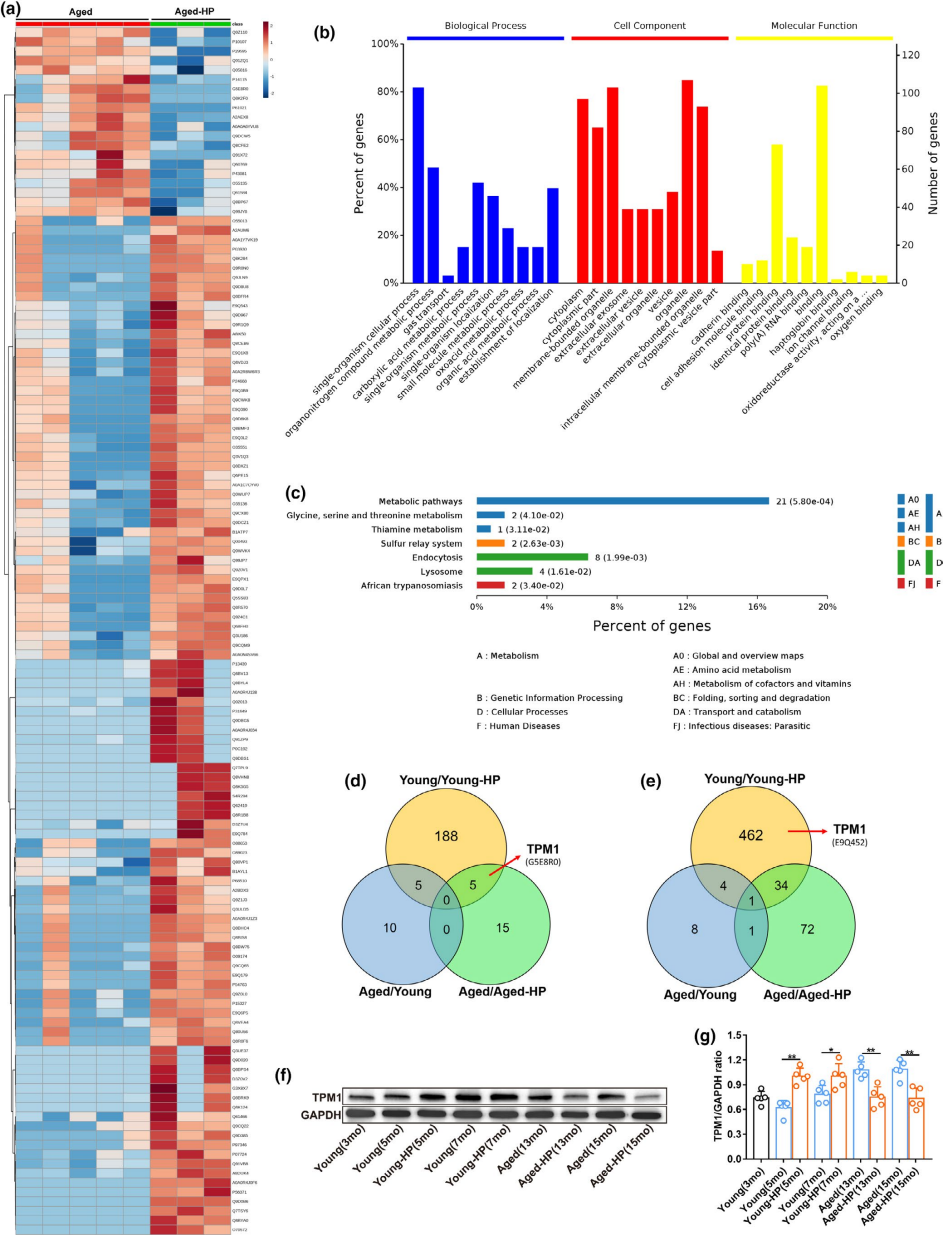
Analysis of differentially expressed proteins by LC‐MS/MS. (a) A heat map showing the up‐ or downregulation of 128 differentially expressed proteins (DEPs) between aged unpaired (13 months) and aged‐HP (13 months) retinas. (b) Gene ontology (GO) enrichment analysis of DEPs between aged unpaired (13 months) and aged‐HP (13 months) retinas. The Y‐axes indicate the percentage (the left axis) and number (the right axis) of DEPs. (c) KEGG pathway enrichment analysis of DEPs between aged unpaired (13 months) and aged‐HP (13 months) retinas. The numbers of DEPs in a specific pathway and corresponding *p*‐values are shown next to each specific bar. (d–e) A venn diagram showing upregulated (d) or downregulated (e) DEPs among young unpaired/young‐HP, aged/aged‐HP, and aged unpaired/young unpaired retinas. (f–g) Validation of TPM1 by western blot analysis (f) and quantification of TPM1 protein (g) in young unpaired, young‐HP, aged unpaired, and aged‐HP retinas 2 and 4 months after parabiosis. Data are presented as mean ± SEM (*n* = 5 mice in each group) and analyzed by one‐way ANOVA with Tukey's multiple comparison test (young vs. young‐HP, ^*^p < 0.05, ^**^p < 0.01; aged vs. aged‐HP, ^**^p < 0.01)

We showed that systemic factors influenced inflammation and neuronal remodeling in the aging retina. To search for such factors, we employed LC‐MS/MS approach to identify differentially expressed proteins (DEPs) in aged unpaired (13 months) and aged‐HP (13 months) retinas 2 months after parabiosis. A total of 128 DEPs, including 20 upregulated and 108 downregulated proteins, were identified between aged unpaired and age‐HP retinas (Figure 3a). The top ten significantly enriched gene ontology (GO) terms based on biological process (BP), cell component (CC), and molecular function (MF) are shown in Figure 3b. The majority of DEPs were associated with cell process (*p* value: 8.91e−08) and metabolic process (*p* value: 9.64e−08) in BP terms, and 76% of them was involved in cytoplasm (*p* value: 7.20e−13) in CC terms. Based on MF, binding (*p* value: 2.43e−05) was the most represented GO term. KEGG analysis identified the metabolism pathway as the most significantly enriched pathway in aged unpaired and aged‐HP mouse retinas (*p* value: 5.80e−04) (Figure 3c), which was highly correlated with metabolic process enriched in the BP analysis (Figure 3b). Meanwhile, a total of 699 DEPs were identified in young unpaired (3 months) and young‐HP (5 month) mouse retinas (Figure S5A). GO enrichment analysis showed that 72% of annotated DEPs was highly related to cytoplasm (*p* value: 8.65e−56) in CC terms (Figure S5B), and binding (*p* value: 1.25e−17) was the most represented GO term based on MF analysis (Figure S5B). The six most significantly enriched pathways were revealed by KEGG analysis in young unpaired and young‐HP retinas (Figure S5C).

By comparing DEPs among aged unpaired/aged‐HP, young unpaired/young‐HP, and aged unpaired/young unpaired groups, we found 5 upregulated (Figure 3d) and 34 downregulated (Figure 3e) proteins in both aged unpaired/aged‐HP and young unpaired/young‐HP groups. Strikingly, we observed that tropomyosin alpha‐1 chain (TPM1), an actin‐binding protein, was significantly upregulated in aged unpaired/aged‐HP groups (Figure 3a,d) but downregulated in young unpaired/young‐HP groups (Figure 3e; Figure S5A), suggesting that blood sharing during parabiosis might affect TPM1 expression. TPM1 has previously been implicated in age‐related diseases, such as Alzheimer's disease and Parkinson's disease (Castaño et al., 2013; Häbig et al., 1832; Hill‐Burns et al., 2016). Therefore, we postulated that TPM1 could be the candidate molecule underlying age‐related alterations in the retina of heterochronic parabionts.

To validate the proteomics results, we performed western blot analysis. Indeed, we found that TPM1 protein was upregulated in aged unpaired (13 months and 15 months) and young‐HP (5 months and 7 months) retinas but downregulated in aged‐HP and young unpaired mouse retinas compared with age‐matched mouse retinas (Figure 3f–g), which were consistent with the proteomics results. Together these results, further indicated that TPM1 was a pro‐aging molecule underlying inflammation, neuronal remodeling, and functional decline in the aging retina.

### 2.4 Treatment by recombinant TPM1 protein induces dendritic outgrowth and impairs visual function in young mice

**FIGURE 4:**
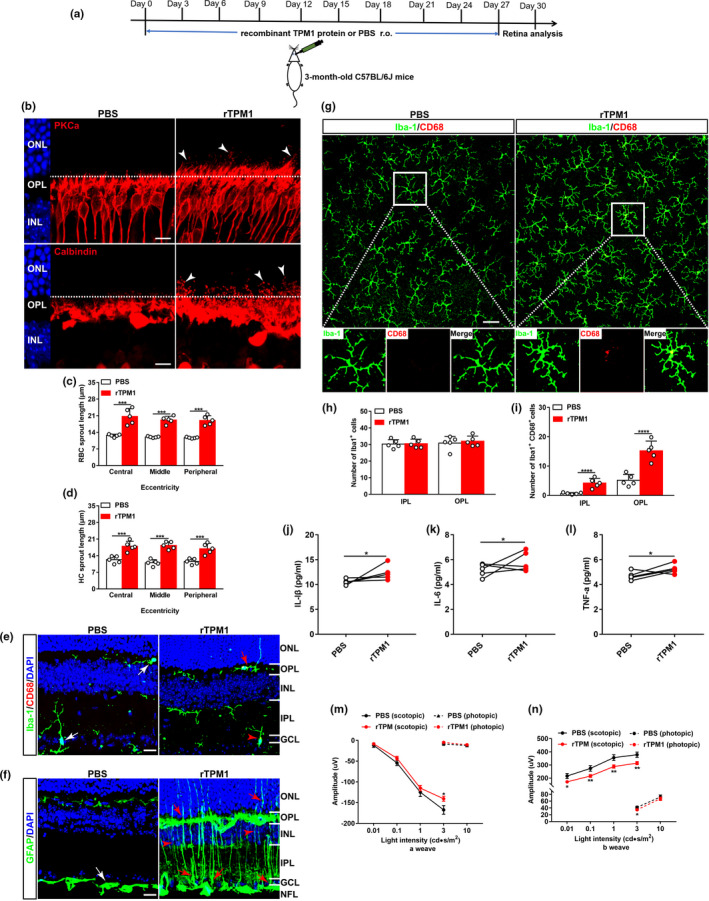
Treatment by recombinant TPM1 protein induces age‐related alternations in young mice. (a) Schematic diagram of the experimental design. Young C57BL/6J mice (3‐month‐old) received a total of ten retro‐orbital injections of recombined TPM1 protein (200 μg/kg), 3 days apart, and retinas were collected for further studies at day 30. (b) Representative confocal images of RBCs (top row) and HCs (bottom row) in retinal sections from young mice after rTPM1 protein or PBS treatment. Arrowheads indicate the aberrant dendritic sprouting of RBCs and HCs. Scale bars, 10 µm. (c–d) Quantification of the aberrant dendritic length of RBCs and HCs in young retinas after rTPM1 protein or PBS treatment. Data are presented as mean ± SEM, *n* = 5 mice in each group, unpaired two‐tailed Student's *t* test. (e) Retina sections stained with Iba‐1and CD68. White arrows show CD68‐negative microglia, red arrowhead indicates CD68‐positive microglia, and red arrow shows the dendritic extension of activated microglia to the ONL. Scale bars, 20 µm. (f) Retinal sections stained with GFAP antibody. White arrow shows resting astrocytes, red arrows indicate activated astrocytes, and red arrowheads show activated Müller cells. Scale bars, 20 µm. (g) Double‐staining of whole‐mounted retinas from young mice after rTPM1 protein or PBS treatment with Iba‐1 and CD68 antibodies. Representative confocal images focus on the OPL. The boxed regions are highly magnified at the bottom showing the colocalization of CD68 with microglia. Scale bar, 50 µm. (h–i) Quantification of the numbers of Iba‐1^+^ and of CD68^+^Iba‐1^+^ microglial cells in the IPL and OPL of whole‐mounted retinas. Data are presented as mean ± SEM, *n* = 4 mice in each group, unpaired two‐tailed Student's *t* test. (j–l) ELISA analysis of IL‐1β, IL‐6, and TNF‐α in retinas from young mice after rTPM1 protein or PBS treatment. Data are presented as mean ± SEM, *n* = 4 mice in each group, unpaired two‐tailed Student's *t* test. (m–n) ERG responses on young mice after rTPM1 protein or PBS treatment. Data are presented as mean ± SEM, *n* = 11 mice in each group, unpaired two‐tailed Student's *t* test

To confirm the pro‐aging role of systemic TPM1, we performed retro‐orbital injections with recombinant TPM1 (rTPM1) protein into young C57BL/6J mice (3 months) (Figure 4a). rTPM1 protein treatment elicited the aberrant dendritic sprouting of RBCs and HCs in young retinas (Figure 4b–d). Additionally, rTPM1 protein treatment triggered microglia activation (Figure 4e–i) and increased the number of activated microglia (CD68^+^Iba1^+^), even though the number of microglia (Iba1^+^) remained unchanged between rTPM1 protein‐treated and PBS‐treated groups (Figure 4g–i). rTPM1 protein treatment also promoted the extension of the dendritic processes of activated microglia into the ONL from the OPL in the young retina (Figure 4e). Similarly, rTPM1 protein treatment increased GFAP immunoreactivity and the incidence of the extension of astrocytic processes to the IPL (Figure 4f). As a result of the glial cell activation, IL‐1β and IL‐6 were elevated in rTPM1 protein‐treated retinas (Figure 4j–l). Functionally, ERG recording showed lower scotopic a‐ and b‐wave amplitudes in rTPM1 protein‐treated mice than in PBS‐treated mice (Figure 4m–n), suggesting functional decline after rTPM1 protein treatment. Together, our results confirm that systemic TPM1 is a pro‐aging factor that influences inflammation, neuronal remodeling, and visual function in the aging retina.

To directly confirm the pro‐aging role of TPM1, we treated BV2 cells with rTPM1 protein. We found that rTPM1 protein treatment activated microglia (CD68^+^) (Figure S6A) and increased the percentage of activated microglial cells (CD45^+^CD11b^+^) (Figure S6B–C), and elevated the expression levels of IL‐1β, IL‐6, and TNFα (Figure S6D–F). Furthermore, we detected both early (Annexin‐V^+^PI^−^) and late (Annexin‐V^+^PI^+^) cell apoptosis in rTPM1 protein‐treated BV2 cells compared with control cells (Figure S6G–I). These in vitro results directly confirm that TPM1 is capable of triggering inflammation and cell apoptosis.

### 2.5 Systemic administration of TPM1‐specific neutralizing antibody counteracts age‐related dendritic outgrowth and visual function decline in aged mice

**FIGURE 5:**
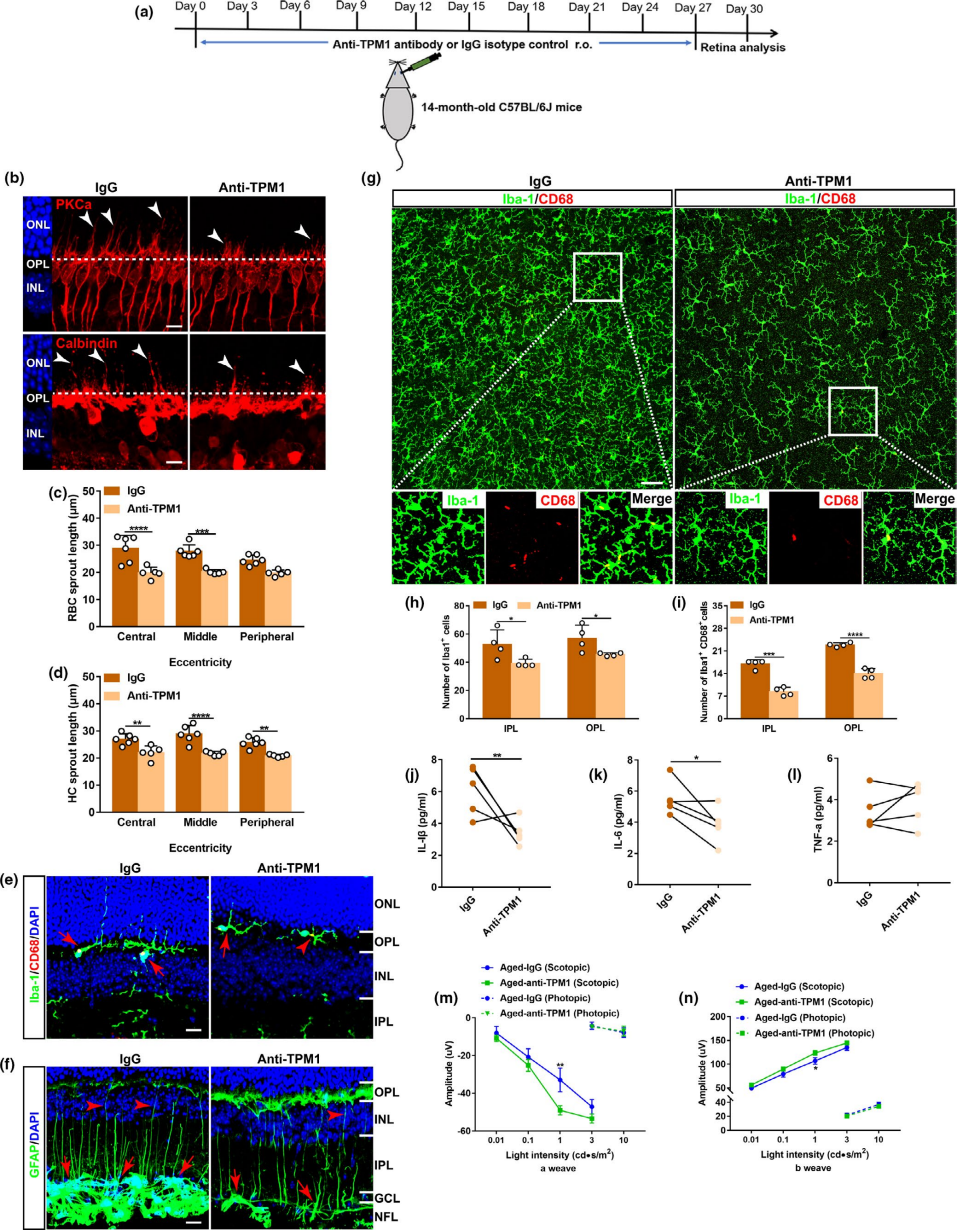
Systemic administration of TPM1‐specific antibody counteracts age‐related dendritic sprouting and inflammation and improves visual function in aged mice. (a) Schematic diagram of the experimental design. Anti‐TPM1 antibody or IgG isotype control (1 mg/kg) were retro‐orbitally injected into aged C57BL/6J mice (14‐month‐old) for a total of ten times, 3 days apart, and retinas were then collected for further studies at day 30. (b) Representative confocal images of RBCs (top row) and HCs (bottom row) in retinal sections from aged mice after anti‐TPM1 antibody or IgG treatment. Arrowheads indicate the aberrant dendritic sprouting of RBCs and HCs. Scale bars, 10 µm. (c–d) Quantification of the aberrant dendritic length of RBCs and HCs in aged retinas after anti‐TPM1 antibody or IgG treatment. Data were presented as mean ± SEM, *n* = 6, 5 mice in IgG and Anti‐TPM1 groups, unpaired two‐tailed Student's *t* test. (e) Retina sections from aged mice after anti‐TPM1 antibody or IgG treatment were stained with Iba‐1and CD68. Red arrowhead indicates CD68‐positive microglia, and red arrows show the dendritic extension of activated microglia. Scale bars, 20 µm. (f) Immunostaining of retinal sections from aged mice after anti‐TPM1 antibody or IgG treatment with GFAP antibody. Red arrows show activated astrocytes, and red arrowheads indicate activated Müller cells. Scale bars, 20 µm. (g) Double‐staining of whole‐mounted retinas from aged mice after anti‐TPM1 antibody or IgG treatment with Iba‐1 and CD68. Representative confocal images focus on the OPL. The boxed regions are highly magnified at the bottom showing the colocalization of CD68 with microglia. Scale bar, 50 µm. (h–i) Quantification of the numbers of Iba‐1^+^ and of CD68^+^Iba‐1^+^ microglial cells in the IPL and OPL of whole‐mounted aged retinas after anti‐TPM1 antibody or IgG treatment. Data are presented as mean ± SEM, *n* = 4 mice in each group, unpaired two‐tailed Student's *t* test. (j–l), ELISA analysis of IL‐1β, IL‐6, and TNF‐α in aged retinas after anti‐TPM1 antibody or IgG treatment. Data are presented as mean ± SEM, *n* = 5 mice in each group unpaired two‐tailed Student's *t* test. (m–n) ERG recordings on aged mice after anti‐TPM1 antibody or IgG treatment. Data are presented as mean ± SEM, *n* = 10 mice in each group, unpaired two‐tailed Student's *t* test

To further confirm the pro‐aging role of systemic TPM1, we suppressed TPM1 expression by performing retro‐orbital injections of TPM1‐specific neutralizing antibody or IgG isotype control antibody into aged C57BL/6J mice (14 months) (Figure 5a). Interestingly, we found that anti‐TPM1 antibody treatment attenuated the aberrant dendritic sprouting of RBCs and HCs in aged mice compared with aged mice receiving IgG isotype control (Figure 5b–d). Additionally, anti‐TPM1 antibody treatment reduced the numbers of microglial cells (Iba1^+^) and of activated microglia (CD68^+^Iba1^+^) (Figure 5g–i) and inhibited the incidence of the extension of the dendritic processes of activated microglia to the ONL from the OPL in aged retinas compared with old mice treated with IgG isotype control (Figure 5e). Similarly, anti‐TPM1 antibody treatment ameliorated GFAP immunoreactivity and decreased the incidence of the extension of astrocytic processes to the IPL from the NFL (Figure 5f). As a result, IL‐1β and IL‐6 were downregulated in aged retinas given anti‐TPM1 antibody (Figure 5j–l). ERG recording showed that anti‐TPM1 antibody treatment enhanced retinal function showing by higher a‐ and b‐wave amplitudes of scotopic ERG relative to old mice treated with IgG isotype controls at 1 cd•s/m^2^ light intensity (Figure 5m–n). These results further confirm that systemic TPM1 influences inflammation, neuronal remodeling, and function in the aging retina.

To further verify the pro‐aging role of systemic TPM1, we specifically removed TPM1 from OMP by immunoprecipitation. We then performed retro‐orbital injections of TMP1‐depleted OMP in young C57BL/6J mice (3‐month‐old) and evaluated its effect on the retina of young mice (Figure S7A–B). Interestingly, we found that TMP1‐depleted OMP failed to induce dendritic sprouting of RBCs and HCs as observed in age‐matched young control mice treated with normal OMP (Figure S7C–E). Similarly, TMP1‐depleted OMP did not cause glial cell activation, which were observed in the retina of age‐matched young control mice treated with normal OMP (Figure S7F–G). Taken together, these findings from our two independent experiments collectively confirm that TPM1 is a systemic pro‐aging factor involved in accelerating retinal aging.

### 2.6 Systematic TPM1 functions by phosphorylating PKA in aging retinas

**FIGURE 6:**
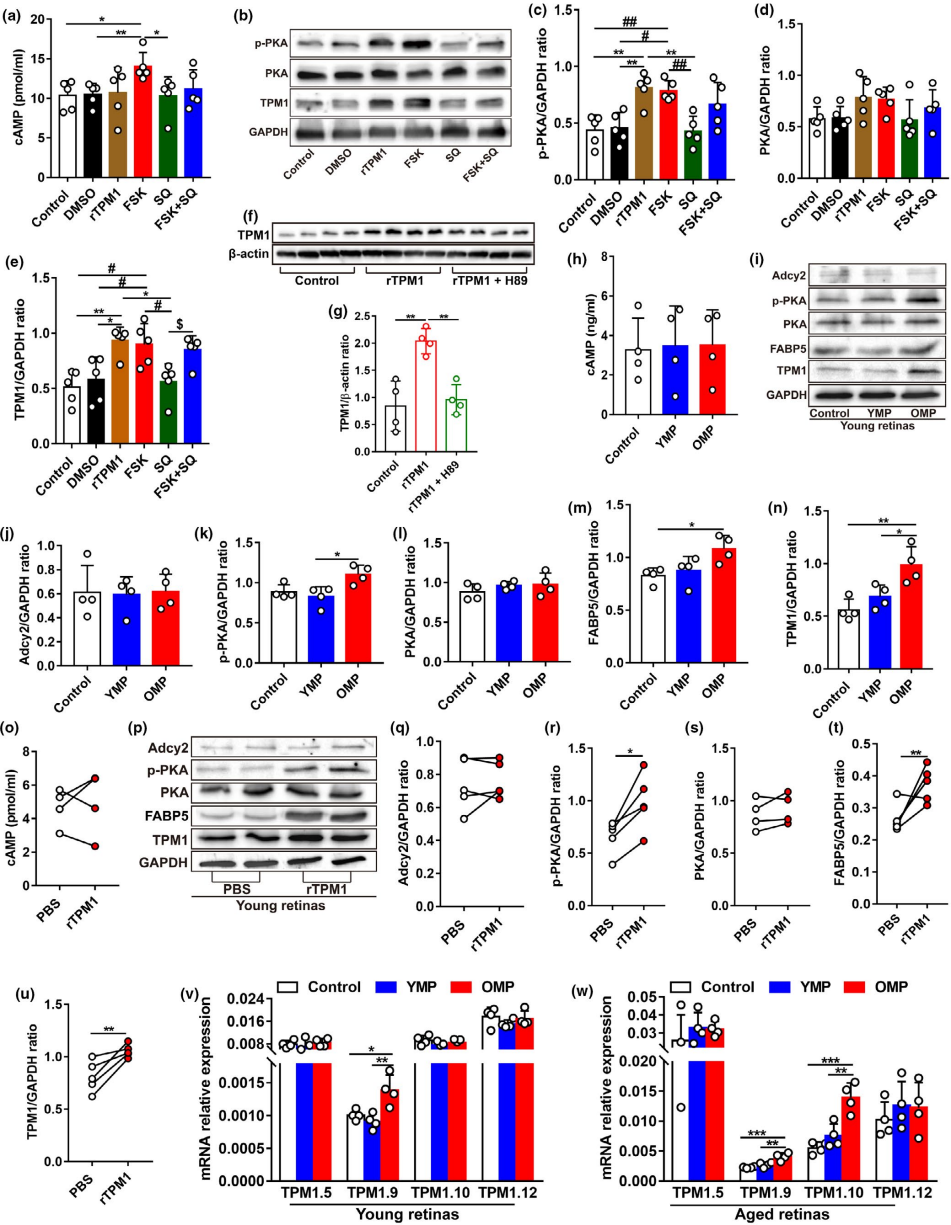
Systematic TPM1 functions by phosphorylating PKA in aging retinas. (a) ELISA analysis of cAMP in BV2 cells after different treatments. Following exposure to DMSO, rTPM1, FSK, SQ, or FSK + SQ for 24 h, BV2 cells were collected to detect the protein level of cAMP. Data are presented as mean ± SEM and analyzed by one‐way ANOVA with Tukey's multiple comparison test (compared to FSK, ^*^p < 0.05). Five independent experiments were performed. (b–e) Western blot analysis (b) and quantification of TPM1, p‐PKA, and PKA (c–e) in BV2 cells after different treatments. Data are presented as mean ±SEM and analyzed by one‐way ANOVA with Tukey's multiple comparison test (compared with rTPM1, ^*^p < 0.05, ^**^p < 0.01; compared with FSK, *^#^p* < 0.05, *^##^p* < 0.01; SQ vs. FSK + SQ, *^$^p* < 0.05). Five independent experiments were performed. (f–g) Western blot analysis (f) and quantification of TPM1 (g) in BV2 cells after rTPM1 and H89 treatments. Data are presented as mean ± SEM and analyzed by one‐way ANOVA with Tukey's multiple comparison test (compared with rTPM1, **p < 0.01). (h) ELISA analysis of cAMP in young retinas after YMP or OMP treatment. Data are presented as mean ± SEM, *n* = 5 mice in each group. (i–n) Western blot analysis (i) and quantification of Adcy2, p‐PKA, PKA, FABP5, and TPM1 (j–n) in young retinas after YMP or OMP treatment. Data are presented as mean ± SEM, *n* = 4 mice in each group, one‐way ANOVA with Tukey's multiple comparison test. (o) ELISA analysis of cAMP in young retinas after rTPM1 protein or PBS treatment. Data are presented as mean ± SEM, *n* = 4 mice in each group. (p–u) Western blot analysis (p) and quantification of Adcy2, p‐PKA, PKA, FABP5, and TPM1 proteins (q–u) in young retinas after rTPM1 protein or PBS treatment. Data are presented as mean ± SEM, *n* = 5 mice in each group, unpaired two‐tailed Student's *t* test. (v–w) qPCR analysis of TPM1.5, TPM1.9, and TPM1.10 in young (v) or aged (w) retinas after YMP or OMP treatment. Data are presented as mean ± SEM, *n* = 4 mice in each group, one‐way ANOVA with Tukey's multiple comparison test

The KEGG pathway analysis revealed that TPM1 was highly associated with adrenergic signaling in cardiomyocytes pathway (*p* value: 2.07e−02) (Figure S5C). Adcy2, a member of adenylyl cyclase (AC), presented significantly differential expression in aged unpaired and aged‐HP mouse retinas (Figure 3a). Therefore, we postulated that TPM1 was potentially related to the AC/cAMP/PKA signal pathway. To confirm our hypothesis, we treated BV2 cells with forskolin (FSK), an activator of AC, and found that cAMP protein was significantly elevated (Figure 6a). Meanwhile, phosphorylated protein kinase A (p‐PKA) and TPM1 proteins were upregulated in BV2 cells treated with FSK (Figure 6b–e). Conversely, treatment by SQ22536 (SQ), an inhibitor of AC, reduced cAMP expression (Figure 6a). p‐PKA and TPM1 proteins were also downregulated in BV2 cells treated with SQ (Figure 6b–e), indicating that AC regulates endogenous TPM1 expression via the AC/cAMP/PKA signaling pathway. Interestingly, we found that rTPM1 protein treatment remarkably upregulated p‐PKA and endogenous TPM1 in BV2 cells (Figure 6b–e). However, we found that rTPM1 protein failed to induce the expression of endogenous TPM1 in BV2 cells treated with H89, an inhibitor of PKA (Figure 6f–g). Together, our data suggest that accumulation of systematic TPM1 may potentially regulate endogenous TPM1 expression by phosphorylating PKA.

Moreover, we observed that the soluble TPM1 protein was significantly higher in OMP or OMS than in YMP or YMS (Figure S8A‐B). Western blotting analysis showed that OMS treatment elicited endogenous TPM1 upregulation in BV2 cells (Figure S8C‐D), which was similar to that in LPS‐treated BV2 cells, suggesting that OMS induced endogenous TPM1 upregulation. Similarly, OMP treatment increased endogenous TPM1 and p‐PKA protein expression levels in the retina of young (Figure 6i,k,n) and aged (Figure S8H,J,M) mice. However, we detected no changes in cAMP, Adcy2, and PKA expression levels in the retina of young (Figure 6h–j,l) and aged (Figure S8G–I, K) mice after OMP treatment, indicating that OMP treatment elevates endogenous TPM1 expression via phosphorylating PKA.

Furthermore, rTPM1 protein treatment induced the upregulation of endogenous TPM1 in BV2 cells (Figure S8E–F) and in the retina of young mice (Figure 6p,u). We also observed no change in Adcy2, cAMP, and PKA expression (Figure 6o–q,s) but in p‐PKA expression (Figure 6p,r) in the retina of young mice after rTPM1 protein treatment. Together, our results confirm that the accumulation of systematic TPM1 induces endogenous TPM1 expression by regulating PKA phosphorylation.

Given that the genes that regulated TPM1 protein expression were different in aged unpaired/aged‐HP and young unpaired/young‐HP retinas (Figure 3d–e), we assumed that different isoforms of TPM1 gene might regulate TPM1 protein expression in young and aged retinas. Therefore, ten known isoforms of TPM1 gene were quantified in BV2 cells after treatment with LPS, which induced TPM1 elevation (Figure S8R–S). qPCR analysis showed that TPM1.5, TPM1.9, and TPM1.10 isoforms were the most significantly upregulated in LPS‐treated BV2 cells (Figure S8T). Similarly, qPCR analysis demonstrated upregulation of TPM1.5, TPM1.9, and TPM1.10 isoforms in OMS‐treated BV2 cells (Figure S8U–W). Meanwhile, OMP treatment induced the mRNA overexpression of TPM1.9 in young retinas (Figure 6v) and of both TPM1.9 and TPM1.10 in aged retinas (Figure 6w), strongly suggesting that TPM1.9 and TPM 1.10 isoforms could be two possible genes regulating endogenous TPM1 protein expression in aging retinas.

### 2.7 Accumulation of systematic TPM1 activates the fatty acid‐binding protein 5 (FABP5)/NF‐κB signal pathway to regulate endogenous TPM1 expression in aging retinas

**FIGURE 7:**
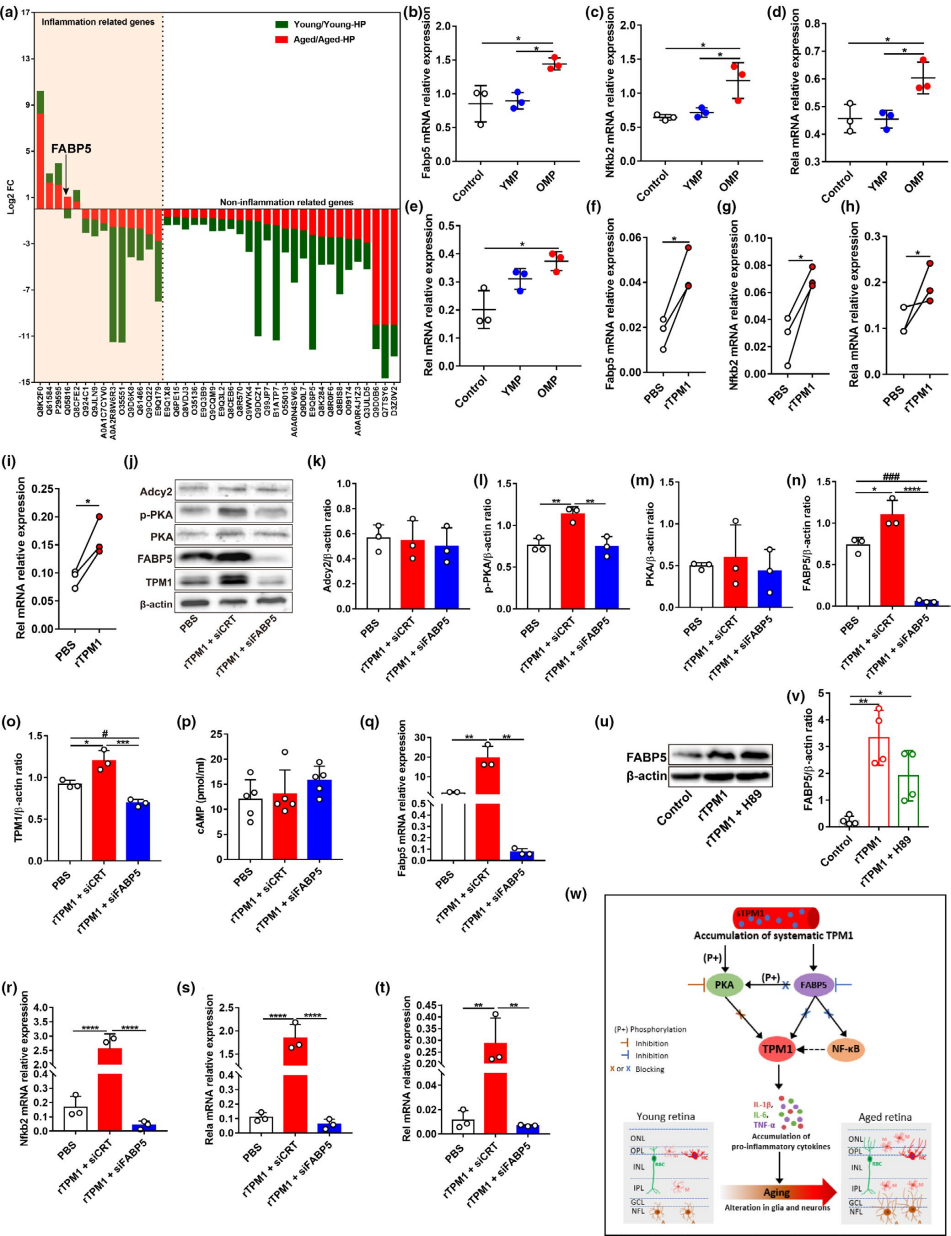
Systematic TPM1 functions through the FABP5/NF‐kB signal pathway. (a) Inflammation‐related DEPs in pairwise comparisons both between young and young‐HP and between aged and aged‐HP. The Y‐axes indicate the Log2 FC of co‐expressed DEPs. (b–e) qPCR analysis of Fabp5, Nfkb2, Rela, and Rel in young retinas after YMP or OMP treatment. Data are presented as mean ± SEM, *n* = 3 mice in each group, one‐way ANOVA with Tukey's multiple comparison test. (f–i) qPCR analysis of Fabp5, Nfkb2, Rela, and Rel in young retinas after rTPM1 protein or PBS treatment. Data are presented as mean ± SEM, *n* = 3 mice in each group, unpaired two‐tailed Student's *t* test. (j–o) Western blot analysis (j) and quantification of Adcy2, p‐PKA, PKA, FABP5, and TPM1 (k–o) proteins in BV2 cells after rTPM1 treatment following the transfection of siFABP5. Data are presented as mean ± SEM and analyzed by one‐way ANOVA with Tukey's multiple comparison test (compared with rTPM1 + siCRT, ^*^p < 0.05, ^**^p < 0.01, ^***^p < 0.001, ^****^p < 0.001; compared with rTPM1 + siFABP5, *^#^p* < 0.05, *^###^p* < 0.001). (p) ELISA analysis of cAMP in BV2 cells after rTPM1 protein treatment following the transfection of siFABP5. Data are presented as mean ± SEM. (q–t) qPCR analysis of Fabp5, Nfkb2, Rela, and Rel in BV2 cells after rTPM1 treatment following the transfection of siFABP5. Data are presented as mean ± SEM, one‐way ANOVA with Tukey's multiple comparison test. Three independent experiments were performed. (u–v) Western blot analysis (u) and quantification of FABP5 (v) in BV2 cells after rTPM1 and H89 treatment. Data are presented as mean ± SEM. (w) Schematic depicting TPM1‐related signaling pathway. Systematic TPM1 accumulation in young‐HP, OMP‐treated or rTPM1 protein‐treated young retina upregulates endogenous TPM1 by phosphorylating PKA and by activating FABP5/NF‐kB signaling pathway, leading to elevated inflammatory responses and neuronal remodeling in retina aging process

By analyzing co‐expressed DEPs between young/young‐HP and aged/aged‐HP, we found that 14 co‐expressed DEPs were related to inflammation (Figure 7a). Among them, FABP5, a fatty acid‐binding protein, was upregulated in aged/aged‐HP mouse retinas (Log2FC: 1.0342) but was downregulated in young/young‐HP mouse retinas (Log2FC: −0.7715), which shows a similar expression trend as TPM1 (Figure 3d–g), suggesting a potential interaction between FABP5 and TPM1. Evidently, we found that FABP5 was overexpressed in OMP‐treated young or aged retinas compared with age‐matched controls (Figure 6i,m; Figure S8H,L), which was consistent with its transcriptome level (Figure 7b; Figure S8N). FABP5 is reported to regulate inflammation via the activation of the NF‐κB signaling pathway (Bogdan et al., 2018; Hui et al., 2010; Senga et al., 2018). After OMP treatment in young or aged mice, we found that the transcriptome levels of three subunits of NF‐κB including Nfκb2, Rela, and Rel were significantly elevated compared with age‐matched controls (Figure 7c–e; Figure S8O–Q), indicating the activation of NF‐κB signaling. Moreover, retro‐orbital injections with rTPM1 protein also elevated the expression levels of FABP5 (Figures 6p,t and 7f) as well as Nfκb2, Rela, and Rel in young mouse retinas (Figure 7g–i), suggesting that systematic TPM1 is capable of triggering the FABP5/NF‐κB signaling pathway in the retina.

To further confirm the interaction between TPM1 and FABP5, we transfected BV2 cells with siFABP5 followed by rTPM1 protein treatment. After FABP5 knockdown following siFABP5 transfection, we found that endogenous TPM1 was significantly downregulated in rTPM1 protein‐treated BV2 cells (Figure 7j,n–o), suggesting that systematic TPM1 potentially activates FABP5 to induce endogenous TPM1 expression. Similarly, we found that the transcriptome levels of Fabp5, Nfkb2, Rela, and Rel were significantly downregulated after FABP5 knockdown in rTPM1 protein‐treated BV2 cells compared with control groups (Figure 7q–t), suggesting that accumulation of systematic TPM1 regulates endogenous TPM1 expression by activating the FABP5/NF‐κB signaling pathway. Interestingly, we observed that FABP5 knockdown did not affect the expression of cAMP, Adcy2, and PKA but p‐PKA in BV2 cells following rTPM1 protein treatment (Figure 7j–m,p), indicating that FABP5 might regulate PKA phosphorylation. Furthermore, we found that rTPM1 protein had no effect on FABP5 expression in BV2 cells after treatment with H89, an inhibitor of PKA (Figure 7u–v), suggesting that PKA is the downstream signaling molecule of FABP5.

Taken together, our data support a model in which systematic TPM1 accumulation phosphorylated PKA and activated FABP5/NF‐κB signaling pathway to induce endogenous TPM1 upregulation (Figure 7w). In addition, the activation of FABP5 also regulated phosphorylation of PKA to affect endogenous TPM1 expression (Figure 7w). Phosphorylated PKA is reported to regulate the transcriptional activities of p65 (Rela), p50, and c‐Rel (King et al., 2011; Oeckinghaus & Ghosh, 2009). Therefore, phosphorylated PKA might also regulate endogenous TPM1 expression by enhancing NF‐κB activity. Consistently, previous studies reported the phosphorylation of PKA affected NF‐κB pathways in regulating inflammatory responses (Ghosh et al., 2015; Oeckinghaus & Ghosh, 2009). Subsequently, endogenous TPM1 upregulation induced the release of pro‐inflammatory cytokines and accelerated the activation of glial cells and neuronal remodeling in aging retinas (Figure 7w).

### 2.8 TPM1 is upregulated in mouse models of Alzheimer's disease and in old human plasma

**FIGURE 8:**
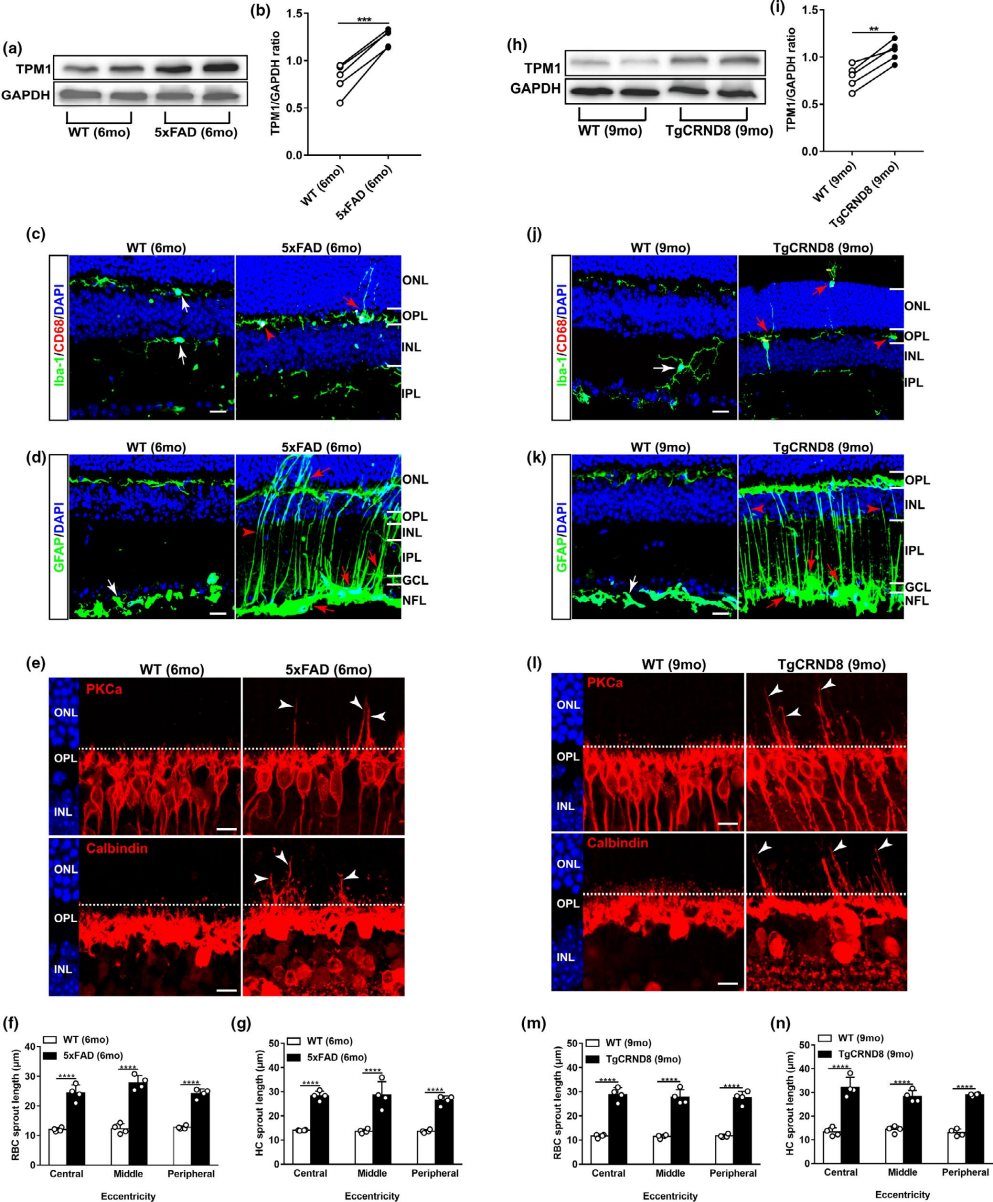
TPM1 upregulation and age‐related retinal alternations in mouse models of Alzheimer's disease. (a–b) Western blot analysis (a) and quantification of TPM1 (b) in C57BL/6J (WT) and 5xFAD retinas. Data are presented as mean ± SEM, *n* = 5 mice in each group, unpaired two‐tailed Student's *t* test. (c) Retina sections from WT and 5xFAD retinas were stained with Iba‐1 and CD68. White arrows indicate CD68^−^ microglia. Red arrowhead shows CD68^+^ microglia, and red arrow indicates the dendritic extension of activated microglia. Scale bars, 20 µm. (d) Retina sections from WT and 5xFAD retinas were stained with GFAP. White arrow shows resting astrocytes. Red arrows show activated astrocytes, and red arrowhead indicates activated Müller cells. Scale bars, 20 µm. (e) Confocal images of RBCs (top row) and HCs (bottom row) in retinal sections from WT and 5xFAD mice. Arrowheads indicate the aberrant sprouting of RBCs and HCs. Scale bars, 10 µm. (f‐g) Quantification of the aberrant dendritic length of RBCs and HCs. Data are presented as mean ± SEM, *n* = 4 mice in each group, unpaired two‐tailed Student's *t* test. (h–i) Western blot analysis (h) and quantification of TPM1 (i) in WT and TgCRND8 retinas. Data are presented as mean ± SEM, *n* = 5 mice in each group, unpaired two‐tailed Student's *t* test. (j) Retina sections from WT and TgCRND8 retinas were stained with Iba‐1 and CD68. White arrow indicates CD68^−^ microglia. Red arrowheads show CD68^+^ microglia, and red arrows indicate the dendritic extension of activated microglia. Scale bars, 20 µm. (k) Retina sections from WT and TgCRND8 retinas were stained with GFAP. White arrow shows the resting astrocytes. Red arrows show activated astrocytes, and red arrowheads indicate activated Müller cells. Scale bars, 20 µm. (l) Confocal images of RBCs (top row) and HCs (bottom row) in retinal sections from WT and TgCRND8 mice. Arrowheads indicate the aberrant dendritic sprouting of RBCs and HCs. Scale bars, 10 µm. (m–n) Quantification of the aberrant dendritic length of RBCs and HCs. Data are presented as mean ± SEM, *n* = 4 mice in each group, unpaired two‐tailed Student's *t* test

Alzheimer's disease (AD) is the most common age‐associated neurodegenerative disorder that slowly leads to cognitive impairment and dementia (Oboudiyat et al., 2013; Soria Lopez et al., 2019). To explore whether TPM1 had a potential role in age‐related neurodegenerative diseases, we examined TPM1 expression in young 5xFAD and TgCRND8 mice, two well‐known rodent AD models (Kim et al., 2019; Poon et al., 2018). Western blot analysis showed that TPM1 was upregulated in 5xFAD and TgCRND8 retinas compared with age‐matched control mice (Figure 8a–b,h–i). Additionally, we observed activated microglia and the extension of the dendritic processes of activated microglia to the ONL from the OPL in both young AD mouse retinas (Figure 8c,j). Similarly, increased GFAP immunoreactivity and the extension of some astrocytic processes into the IPL from the NFL were observed in the two young AD mouse models (Figure 8d,k). Interestingly, the two young AD mouse models developed a similar degree of dendritic sprouting in RBCs and HCs in the young retina as the normal aging retina (Figure 8e–g,l–n). Together our results indicate that elevated TPM1 potentially contributes to inflammatory responses and the dendritic sprouting of RBCs and HCs in the young AD mouse models. These results suggest a possible critical role of TPM1 in age‐related neurodegenerative diseases.

Moreover, we explored whether TPM1 displayed a similar age‐related increase in aged human samples. To this end, we measured TPM1 protein expression levels in human blood plasma from both young and aged donors (Table S2). Interestingly, we found that TPM1 protein level was significantly higher in blood plasma from aged donors than in those from young donors (Figure S8X), suggesting that TPM1 expression changes with age in humans. Taken together, these results indicate that TPM1 could contribute to aging process.

## 3 DISCUSSION

In this study, we uncovered a new role for the actin‐associated protein tropomyosin 1 (TPM1) in regulating inflammation and neuronal remodeling in the aging retina. We demonstrated that inflammatory responses, the aberrant dendritic sprouting of rod bipolar (RBC) and horizontal (HC) cells and visual function decline were accelerated in young heterochronic parabionts or young mice treated with old mouse plasma (OMP). Conversely, old heterochronic parabionts or old mice treated with young mouse plasma (YMP) showed the attenuation of inflammatory responses and the dendritic outgrowth of RBCs and HCs and of visual function decline. These results confirmed that systemic factors regulated age‐related inflammation and neuronal remodeling in the aging retina. Proteomic analysis revealed that TPM1 was a potential systemic molecule underlying structural and functional deficits in the aging retina. Retro‐orbital administration of recombinant TPM1 protein in young mice showed similar detrimental effects on the retina as young heterochronic parabionts or young mice treated with OMP, whereas retro‐orbital injection of anti‐TPM1 neutralizing antibodies in aged mice reproduced the beneficial effects on the retina as old heterochronic parabionts or old mice treated with YMP. Moreover, retro‐orbital injection of TMP1‐depleted OMP in young mice failed to reproduce age‐related detrimental effects on the retina as observed in young mice treated with normal OMP. Collectively, our data confirmed that TPM1 was a systemic pro‐aging factor that was involved in accelerating retina aging in the central nervous system.

Our *in vitro* and *in vivo* experiments consistently demonstrated that systematic TPM1 was an immune‐related molecule, which elicited endogenous TPM1 expression by phosphorylating PKA and activating the FABP5/NF‐κB signaling pathway in aging retinas (Figure 7w). Similar to TPM1, we found that FABP5 was upregulated in pairwise comparisons between aged and aged‐HP mouse retinas and downregulated in pairwise comparisons between young and young‐HP mouse retinas, suggesting a potential interaction between TPM1 and FABP5 in aging retinas. FABP5 knockdown significantly downregulated endogenous TPM1 and the NF‐κB signaling pathway, indicating that FABP5 regulated endogenous TPM1 expression via the NF‐κB signaling pathway. Interestingly, we found that FABP5 regulated endogenous TPM1 expression by phosphorylating PKA. Indeed, FABP5 is reported to regulate the endocannabinoid (eCB) signals in synaptic transport (Haj‐Dahmane et al., 2018). The activation of CB1Rs (composed of cannabinoid receptor type 1), which is regulated by eCBs, affects the cAMP/PKA signaling pathway to modulate synaptic plasticity in the brain (Chevaleyre et al., 2007; Haj‐Dahmane & Shen, 2010; Herkenham et al., 1991), suggesting that FABP5 might regulate the cAMP/PKA pathway. Collectively, endogenous TPM1 upregulation subsequently drove inflammatory responses and the dendritic sprouting of RBCs and HCs and visual function decline in the aging retina (Figure 7w). TPM1 is reported to regulate neurite outgrowth and cell migration (Bach et al., 2009; Curthoys et al., 2014). It thus remains possible that age‐related elevation of endogenous TPM1 could potentially drive the dendritic sprouting of RBCs and HCs directly in the aging retina (Figure 7w).

Previous studies reported that the serine/threonine kinase LKB1 regulated age‐related synaptic remodeling and axon retraction in rod photoreceptors (Samuel et al., 2014), which form synapses with RBCs and HCs in the outer retina. Yet, the molecular mechanisms underlying the dendritic sprouting of RBCs and HCs in aging retinas remain unknown. In this study, we found that TPM1 overexpression accelerated the dendritic sprouting of RBCs and HCs in young retinas and TPM1 inhibition suppressed the dendritic sprouting in old retinas, suggesting that systematic TPM1 was the molecular basis underlying the dendritic sprouting of RBCs and HCs in aging retinas. We further demonstrated that systematic TPM1 accumulation elevated endogenous TPM1 in aging retinas to accelerate the dendritic outgrowth of RBCs and HCs by phosphorylating PKA and activating the FABP5/NF‐κB pathway. However, it remains unclear how LKB1 and TPM1 coordinate with each other in regulating axon retraction in rods and dendritic sprouting in RBCs and HCs, respectively.

We showed that an age‐related accumulation of systemic TPM1 in aged mice was responsible for endogenous TPM1 elevation, the dendritic sprouting of RBCs and HCs, and functional decline in the aging retina. However, it remains unclear how exactly elevated TPM1 in circulation conveys its pro‐aging signals to influence endogenous TPM1 expression in the aging retina, which is separated anatomically by the blood–retinal barrier that tightly regulates the movement of molecules between the blood and the retina (Campbell & Humphries, 2012; Runkle & Antonetti, 2011). Previous parabiosis studies have consistently demonstrated a robust connection between systemic circulating factors in blood and the brain (Liu et al., 2017; Smith et al., 2018). Several routes are reported to convey information from the periphery to the brain (Liu et al., 2019; Salvador et al., 2021; Satoh et al., 2017). For instance, vascular cell adhesion molecule 1 expressed by brain endothelial cells, which binds to leukocytes at the blood–brain barrier, has been reported to relay pro‐aging signals from the blood to the brain (Yousef et al., 2019). However, it is unclear whether retinal endothelial cells that form the blood–retinal barrier are similarly involved in the transmission of pro‐aging TPM1 signals from the periphery to the aging retina. Additional experiments will be needed to address the possibility.

Interestingly, we found that the two common mouse models of Alzheimer's disease (AD) developed retinal deficits including increased inflammatory responses and the ectopic dendritic sprouting of RBCs and HCs at young age similar to those that occurred in the normal aging retina. In addition, the two young AD mouse models all exhibited the significant upregulation of TPM1 in the retina as well, which was observed in the normal aging retina. Consistently, TPM1 upregulation was reported in the white matter of AD patient brains (Castaño et al., 2013). Our findings suggest that TPM1 might potentially play a similar role in age‐related neurodegenerative diseases as in normal brain aging. However, further investigations are needed to validate this hypothesis.

In conclusion, we demonstrated that systemic TPM1 accumulation promoted age‐related TPM1 elevation, inflammatory responses, neuronal remodeling, and functional decline in the aging retina via the modulation of the FABP5/NF‐κB pathway and the PKA phosphorylation. These results suggest therapeutical strategies for attenuating these age‐related retinal defects by targeting TPM1 in old age. Interestingly, we observed that TPM1 showed an age‐related accumulation in human blood plasma, indicating that TPM1 could be a potential biomarker for aging. Considering that the conclusion of this study is limited to aging mice, further investigation of its therapeutic potential for aging individuals and those suffering from age‐related neurodegenerative diseases is warranted.

## 4 MATERIAL AND METHODS

### 4.1 In vivo experiments

#### 4.1.1 Mice

C57BL/6J (Stock no: 000664) and Cx3cr1^GFP/GFP^ (Stock no: 021160) mice were obtained from Jackson Laboratory. 5xFAD mice on C57BL/6J background (https://www.alzforum.org/research‐models/5xfad‐c57bl6) and TgCRND8 mice on Hybrid C3H/He‐C57BL/6J background (https://www.alzforum.org/research‐models/tgcrnd8), two commonly used AD transgenic mouse models of Alzheimer's disease (AD), were generously provided by Dr. Youqiang Song at University of Hong Kong. All mice were housed in a 12‐h light/dark cycle with water and food *ad libitum* and maintained at the Centralised Animal Facilities, The Hong Kong Polytechnic University. C57BL/6J mice were backcrossed with Cx3cr1^GFP/GFP^ mice, and the littermates from C57BL/6J/Cx3cr1^+/GFP^ were used for the confirmation of blood chimerism after parabiosis surgery. C57BL/6J, 5xFAD and TgCRND8 mice of both sexes were used for this aging study. The specific ages of mice were indicated in individual Figures, Figure legends and Methods. All experimental procedures were approved by the Animal Subjects Ethics Sub‐committee (ASESC) of The Hong Kong Polytechnic University and conducted in accordance with the ARVO statement for the use of animals.

#### 4.1.2 Parabiosis surgery

Parabiosis surgeries were performed as previously described by others (Kamran et al., 2013; Yuan et al., 2015). Only female C57BL/6J mice were used for parabiosis surgery. In brief, two female C57BL/6J mice, either young and young (3‐month‐old), old and old (11‐month‐old), young (3‐month‐old) and old (11‐month‐old) or Cx3cr1^+/GFP^ (3‐month‐old) and C57BL/6J mice (3‐month‐old) were housed together to form cohabitation harmony for 2 weeks before the surgery. After anesthesia with a mixture of ketamine hydrochloride (100 mg/kg) and xylazine (20 mg/kg), a mirror‐image skin incision was made on the opposite side of the two mice from the elbow to the knee. Afterward, the elbows and knee joints of animals were connected by non‐absorbable sutures and the skin openings of the adjacent parabionts were seamed together. Following intraperitoneal injection with 0.5 ml normal saline solution and buprenorphine (0.1 mg/kg), parabiotic pairs were put on a heating pat to maintain the body temperature at 37℃ until recovery. For the confirmation of blood chimerism after 2 weeks of the surgery, 3–4 drops of blood from each tail of parabionts were collected in 50 mM EDTA in PBS, followed by adding 5 ml ACK buffer to lyse red blood cell on ice for 5 min. After centrifugation, cell pellets were dissolved in PBS and rinsed for three time and then were resuspended in PBS for flow cytometry analysis.

#### 4.1.3 Mass spectrometry

Retinas from young (3‐month‐old) and aged (13‐month‐old) C57BL/6J mice, young heterochronic parabionts (young‐HP, 5‐month‐old), and aged heterochronic parabionts (aged‐HP, 13‐month‐old) were collected for proteomics analysis. The retina was lysed using the ultrasonic extraction in pre‐cooling lysis buffer for 10 min. After centrifugation at 14,000 *g* for 14 min at 4℃, the supernatant was transferred to a new tube for quantification of protein concentration with Pierce™ rapid gold BCA protein assay kit (Invitrogen). Following the reduction with dithiothreitol (DTT) in NH_4_HCO_3_ at 37℃ in water bath for 3 h and alkylation with iodoacetamide (IAA) in NH_4_HCO_3_ for 1 h at room temperature in dark, proteins were digested with sequencing‐grade trypsin at 37℃, overnight. After desalting and vacuum drying, peptides were dissolved in 0.1% formic acid and separated using Acclaim™ PepMap™ 100 C18 HPLC Columns (Thermo Scientific, USA). The HPLC system was coupled to an Orbitrap Fusion Lumos Tribrid Mass Spectrometer (Thermo Scientific, USA). MS1 survey scans (m/z 350–1,800) were performed at a resolution of 30,000 with a 4,000,000 AGC target. Peptide precursors with charge state ≥2 were sampled for MS2, in which AGC target was set to 20,000 and the maximum injection time was 80 msec, and up to 20 most abundant ions detected in an Orbitrap full MS spectrum were selected for further MS/MS analysis. For data analysis, all raw files from MS were processed with MaxQuant computational proteomics platform (Version 1.5.2.8) with Uniprot‐Mus musculus database, and searching parameters were set as follows: Fixed modification was carbamidomethyl (C); variable modifications was oxidation (M); enzyme was trypsin; maximum missed cleavages was two; peptide mass tolerance and fragment mass tolerance were 20 ppm and 0.6 Da. Differentially expressed proteins were selected with fold change ratio ≥1.5 or ≤0.67 above the 95% confidential level in each comparison. Gene Ontology (GO) annotation and Kyoto Encyclopedia of Genes and Genomes (KEGG) pathway analysis for all differentially expressed proteins were analyzed using the online OmicsBean resource (http://www.omicsbean.cn/).

#### 4.1.4 Plasma and serum preparation from young and old C57BL/6J mice

Mice were anesthetized by 2.5% isoflurane, and the hair around both eyes was shaved, and the skin around two eyes was cleaned by alcohol pads. Eyeballs were completely pushed out by pressing the edges of eyes with scissors, followed by quickly enucleating the eyeballs and the blood came out from the retro‐orbital plexuses. Blood was collected in an EDTA‐coated tube, and the blood sample was centrifugated at 850 x g for 10 min, 4℃. Afterward, the supernatant was transferred into a new sterile centrifuge tube with a filter unit of 0.22 μm, and the sample was spinned down at 2370 x g for 1 min at 4℃ to collect the filtered plasma. For serum collection, blood was collected in a regular sterile tube and put it in room temperature for 20–30 min. Next, the blood was centrifugated and the supernatant was filtered with same conditions to make the filtered serum. Finally, both plasma and serum were individually transferred into the new sterile tubes for analysis or stored at −80℃ for future use. Mouse tropomyosin 1(alpha) ELISA kit (MyBioSource, MBS9325448) was applied to quantify soluble TPM1 protein levels in plasma and serum.

#### 4.1.5 Human blood samples

Young human blood samples were collected from healthy male and female donors in Hong Kong, and the methods of human plasma preparation were similar to what we mentioned for mouse plasma preparation. Aged human plasma was kindly provided by Dr. Youqiang Song and Prof. Leung‐Wing Chu at the Hong Kong University. The age and sex for each donor are listed in Table S2.

#### 4.1.6 In vivo plasma, recombinant TPM1 protein, and anti‐TPM1 antibody injection

For plasma injections, young (3‐month‐old) and aged (14‐month‐old) C57BL/6J mice were retro‐orbitally injected with 150 μl pooled plasma from young (3‐month‐old) or aged (15‐month‐old) mice for a total of ten injections, 3 days apart. For recombinant TPM1 protein injection, young C57BL/6J mice (3‐month‐old) received a total of ten retro‐orbital injections of recombinant TPM1 protein (Novus, MBP1‐48329) at 200 μg/kg over a month. Meanwhile, the same volume of PBS was injected to young C57BL/6J mice (3‐month‐old) in control groups. For antibody injections, anti‐TPM1 antibody (ABclonal, A1157) or IgG isotype control (1 mg/kg) were retro‐orbitally injected to aged C57BL/6J mice (14‐month‐old) every 3 days for a total of ten times.

#### 4.1.7 Immunodepleting TPM1 in old mouse plasma

Old mouse plasma collected from aged mice (15‐month‐old) was incubated with IgG isotype control (1:100) or TPM1 antibody overnight at 4℃ in a rotary mixer. After incubation with 30 µl of protein A/G Sepharose^®^ beads slurry (Abcam) at 4℃ for 1 h, the antibody‐antigen complexes were discarded by centrifugation at 200 *g*, 4℃ for 2 min; the supernatants were collected for further use. The efficiency of TPM1 depletion in old mouse plasma was evaluated by mouse tropomyosin 1 (alpha) ELISA kit (MyBioSource).

#### 4.1.8 Immunocytochemistry and confocal imaging

Animals were anesthetized with a mixture of ketamine hydrochloride (100 mg/kg) and xylazine (20 mg/kg), and eyeballs were quickly enucleated, and the retina was separated from vitreous and sclera in PBS. Afterward, the retina was fixed in 4% paraformaldehyde (PFA) for 1 h and then dehydrated in 30% sucrose overnight at 4℃. Some of retinas were serially sectioned with the thickness of 14 µm on a cryostat microtome. After rinsing for several times and blocking, the cross sections were incubated with PKCa (Santa Cruz, 1:500), calbindin (Swant, 1:500), GFAP (Dako, 1:500), Iba‐1 (Wako, 1:500) or CD68 (Bio‐rad, 1:500) antibodies in blocking buffer, overnight. Secondary antibodies including goat anti‐rabbit Alexa Flour 488 (Invitrogen, 1:500), goat anti‐rat Alexa Fluor 568 (Invitrogen, 1:500), goat anti‐rabbit Alexa Fluor 594 (Invitrogen, 1:500), or goat anti‐mouse Alexa Fluor 594 (Invitrogen, 1:500) were applied to the sections for 2 h before mounting with Dako fluorescence mounting medium. For whole‐mounted retinas, two primary antibodies, Iba‐1 (Wako, 1:500) and CD68 (Bio‐rad, 1:500) in the blocking buffer, were applied for 1 day, followed by secondary goat anti‐rabbit Alexa Flour 488 (Invitrogen, 1:500) and goat anti‐rat Alexa Fluor 568 (Invitrogen, 1:500) antibodies for 2‐h incubation. Imagines were captured by a Zeiss LSM 800 Upright Confocal Microscope (Zeiss, USA) with a pixel resolution of 1,024 × 1,024, and Plan‐Apochromat 40×/1.3 oil‐immersion or 20×/0.8 objectives. Z‐stack images with an interval of 0.5 µm was acquired. For quantification of the aberrant dendritic length of rod bipolar cells or horizontal cells, three regions in each retinal section at 100 µm (central), 1 mm (middle), and 1.8 mm (peripheral) from the optical nerve head were captured, and the distance between the tip of dendrites and cell soma was measured with Image J software. For quantification of microglial cells, four sampling areas with 638.9 µm × 638.9 µm squares along the dorsal–ventral axis of retinal whole mounts at 200 µm and 1 mm from the optic nerve head on both sides were photographed, and the numbers of Iba‐1^+^ and of CD68^+^Iba‐1^+^ cells were manually counted.

#### 4.1.9 Immunoblotting

Total proteins were extracted with T‐PER™ Tissue Protein Extraction Reagent (Invitrogen), and protein was quantified with Pierce™ rapid gold BCA protein assay kit (Invitrogen). After denaturing by boiling in SDS sample buffer, an aliquot of total proteins with thirty micrograms was subjected to 10% SDS‐PAGE gels, followed by transferring to a polyvinylidene difluoride membrane. After blocking for 1 h, the membranes were incubated with primary antibodies at the designated concentrations: rabbit anti‐GFAP (Dako, 1:500), rabbit anti‐Iba‐1 (Wako, 1:500) or rat anti‐CD68 (Bio‐rad, 1:500), rabbit anti‐TPM1 (ABclonal, 1:1,000; Invitrogen, 1:1,000), rabbit anti‐ADCY2 (Abcam, 1:1,000; ABclonal, 1:1,000), rabbit anti‐Phospho‐PKA C (Thr197) (Cell Signaling Technology, 1:1,000), rabbit anti‐PKA C‐α (Cell Signaling Technology, 1:1,000), rabbit anti‐ FABP5 (D1A7T) (Cell Signaling Technology, 1:1,000), or mouse anti‐GAPDH (Sigma‐Aldrich,1:1,000; ABclonal, 1:1,000), overnight at 4℃. Afterward, the membranes were incubated with secondary antibodies including goat anti‐rabbit IgG and goat anti‐mouse IgG conjugated to horseradish peroxidase (Invitrogen, 1:1,000) for 2 h. After incubation with SuperSignal™ West Pico PLUS Chemiluminescent Substrate (Invitrogen) or ECL™ Select Western Blotting Detection Reagent (AmershamTM), the membrane with protein was evaluated by ChemiDoc Imaging Systems (Bio‐rad, California, USA). The optical density value of each brand was measured using Image J software.

#### 4.1.10 Quantitative Real‐Time PCR

After plasma or recombinant TPM1 protein treatment, retinas from mouse eyes were collected. Total RNA was extracted with TransZol Up Plus RNA Kit (TransGen, China), and cDNA was synthetized using the TransScript^®^ First‐Strand cDNA Synthesis SuperMix (TransGen, China). QPCR was performed with PerfectStart™ Green qPCR SuperMix (TransGen, China) using QuantStudio™ 7 Flex Real‐Time PCR System (Applied Biosystems™). GAPDH was used as a control, and the data were analyzed using 2^−ΔCt^ method. Gene‐specific primers used for qPCR are listed in Table S1.

#### 4.1.11 Enzyme‐linked immunosorbent assay (ELISA)

For the detection of inflammatory cytokine TNF‐a, IL‐1β, and IL‐6 in the retina, mouse TNF alpha Elisa Kit (Invitrogen), mouse IL‐1 beta Elisa Kit (Invitrogen), and mouse IL‐6 Elisa Kit (Invitrogen) were used based on the manufacturer's instructions. Optical density was measured at 450 nm on microplate reader (Azure Biosystems). The level of cAMP in the retina after plasma or recombinant TPM1 (rTPM1) protein treatment was evaluated with Cyclic AMP Competitive ELISA Kit (Invitrogen) or Camp ELISA Kit (Mouse) (Aviva Systems Biology). The expression levels of Cox‐1 and Cox‐2 in the retina were measured after rTPM1 protein treatment using mouse COX1 ELISA Kit (MyBioSource) and mouse COX2 ELISA Kit (Abcam), respectively.

#### 4.1.12 Electroretinographic (ERG) analysis

ERGs were performed using a Celeris ERG system (Diagnosys, USA) as previously described by others (Movsas et al., 2018). In brief, mice were intraperitoneally anesthetized with a mixture of ketamine hydrochloride (100 mg/kg) and xylazine (20 mg/kg) following overnight dark adaption, and the pupils were dilated with 1% mydriacyl (Alcon). Mouse was put on a platform heater with temperature set at 37℃, and 3% Hypromellose lubricating gel solution was applied in both corneas and the cups of the electrodes, followed by symmetrically placing electrodes on both eyes. A TOUCH/TOUCH protocol was adopted to detect two eyes, and unstimulated eye was used as the reference. A scotopic ERG was performed on dark‐adapted mice with gradually increasing light intensities from 0.01, 0.1, 1 to 3 cd.s/m^2^, and a photopic ERG was recorded at 3 and 10 cd.s/m^2^ light intensities after 10‐min light adaptation with background light intensity at 30 cd.s/m^2^. Sweep pre‐trigger and post‐trigger times were individually set to 50 and 300 msec, and the sample frequency was 2000 Hz, and ten sweeps were acquired with each light stimulus. The amplitude of ERG a‐wave was measured between the baseline and the negative peak, and the amplitude of b‐wave was measured between the bottom of the a‐wave and the top of the tallest curve.

### 4.2 In vitro experiments

#### 4.2.1 Cell culture

BV2, a murine microglial cell line, was kindly provided by Prof. Haiwei Xu at Southwest Eye Hospital, Chongqing, China (Li et al., 2016). BV2 cells were cultured in DMEM with 10% FBS, 1% penicillin/streptomycin, and 2 mM l‐Glutamine in a 37℃ incubator under 5% CO_2_. BV2 cell was seeded in 6‐well plate (5 × 10^5^ cells per well) or 12‐well plate (5 × 10^4^ cells per well). After starvation with serum‐free medium for 2 h, cells were treated with either 0.1% DMSO, LPS (1 μg/ml), DMEM with 10% serum from young (3‐month‐old) or aged (15‐month‐old) mice, recombinant TPM1 protein (50 ng/ml and 100 ng/ml), forskolin (FSK, 50 μm), H89 (10 µM) or SQ22536 (SQ, 100 μm) for 24 h. Cells were then collected for further experiments.

#### 4.2.2 siRNA transfection

Three siRNAs targeting Fabp5 were designed and synthesized from Synbio Technologies (Suzhou, China). To evaluate the knockdown efficiency of Fabp5, three siRNAs were mixed together and transfected with Lipofectamine^®^ RNAiMAX Reagent (Life Technologies) on BV2 cells. After 2 days, cells were collected to analyze the Fabp5 expression level by qRT‐PCR. For the study of TPM1‐related signaling pathway, cells were transfected with three siFABP5 mixtures or siRNA negative control (provided by Synbio Technologies) for 24 h before treatment with recombinant TPM1 protein (100 ng/ml) for 1 day and then cells were collected for further analysis. Detailed sequences for all siRNAs are listed in Table S1.

#### 4.2.3 Flow cytometry

After different treatments, cells were detached with 0.25% trypsin‐EDTA for 5 min, and the reaction was stopped by resuspending with fresh culture medium. Following washing with PBS for three times, cells were blocked with Fc blocking antibody in staining buffer for 10 min. Afterward, cells were incubated with fluorescent isothiocyanate (FITC)‐conjugated anti‐CD45 and phycoerythrin (PE)‐conjugated anti‐CD11b in staining buffer for 30 min to 1 h at room temperature on a benchtop shaker. Cells were sorted using BD FACSVia Flow Cytometer (BD Biosciences, Franklin Lakes, USA), and data were analyzed by BD FACSVia Research Software. For cell apoptosis detection, both medium and detached cells were collected after different treatments. Following centrifugation at 210 x g for 5 min, cells were rinsed with PBS by gently shaking and were then resuspended in binding buffer to adjust cell density with 2–5 × 10^5^/ml. Following staining with FITC‐conjugated Annexin‐V in binding buffer for 10 min at room temperature, cells were washed again and resuspended in binding buffer with propidium iodide (PI), and then cells were directly detected by BD FACSVia Flow Cytometer, and data were analyzed by BD FACSVia Research Software. All experimental procedures were strictly performed according to the user guide of the apoptosis detection kit.

#### 4.2.4 Immunoblotting

BV2 cells were disassociated with 0.25% trypsin and collected in microcentrifuge tubes. After rinsing three times with PBS, cells were resuspended in RIPA lysis buffer (Abcam) with cocktails of protease and phosphatase inhibitors (Roche). Cell lysates were maintained on ice for 15–20 min, and then samples were centrifugated with 15,000 rpm for 15 min, at 4℃. The supernatants were collected in a new microcentrifuge tube, and the protein concentration was detected with Pierce™ Rapid Gold BCA Protein Assay Kit. Protein samples were denatured by boiling in SDS sample buffer. Thirty micrograms of protein were then subjected to 10% SDS‐PAGE gels and transferred to a polyvinylidene difluoride membrane. After blocking for 1 h, proteins were probed with rabbit anti‐TPM1 (ABclonal, 1:1,000), rabbit anti‐Phospho‐PKA C (Thr197) (Cell Signaling Technology, 1:1,000), rabbit anti‐PKA C‐α (Cell Signaling Technology, 1:1,000), rabbit anti‐ FABP5 (D1A7T) (Cell Signaling Technology, 1:1,000), mouse anti‐GAPDH (Sigma‐Aldrich,1:1,000), or mouse anti‐actin (ABclonal, 1:2,000). Goat anti‐rabbit IgG and goat anti‐mouse IgG (Invitrogen, 1:1,000) conjugated to horseradish peroxidase were applied as secondary antibodies. After incubation with SuperSignal™ West Pico PLUS Chemiluminescent Substrate (Invitrogen), the membrane with protein was evaluated by ChemiDoc Imaging Systems (Bio‐rad, California, USA). The optical density value of each brand was measured using Image J software.

#### 4.2.5 Quantitative Real‐Time PCR

Extraction of total RNA from cell pellets and amplification of cDNA were similar with above. Real‐time PCR was conducted with PerfectStart™ Green qPCR SuperMix (TransGen, China). The reactions were performed by QuantStudio™ 7 Flex Real‐Time PCR System (Applied Biosystems™). GAPDH was used as a control, and data were analyzed using 2^−ΔCt^ method. Gene‐specific primers used for qPCR are listed in Table S1.

#### 4.2.6 Immunocytochemistry and confocal imaging

After removing the culture medium, cells on coverslips were washed with PBS for three times. 4% paraformaldehyde (PFA) solution was added to fix cells for 20 min at room temperature. Following rinsing for three times, cells were incubated with rabbit anti‐Iba‐1 (Wako, 1:500) and rat anti‐CD68 (Bio‐rad, 1:500) in blocking buffer, overnight. Goat anti‐rabbit Alexa Fluor 488 (Invitrogen, 1:500) and goat anti‐rat Alexa Fluor 568 (Invitrogen, 1:500) were applied as secondary antibodies. Coverslips were mounted with Dako fluorescence mounting medium. Imagines were captured by a Zeiss LSM 800 Upright Confocal Microscope (Zeiss, USA). Z‐stack images were acquired at 0.5‐µm intervals using a 20× objective. An orthogonal projection was made to compose a final image.

#### 4.2.7 Enzyme‐linked immunosorbent assay (ELISA)

Total proteins from cell pellets after different treatments were extracted and quantified with BCA kit (Invitrogen). Tumor necrosis factor alpha (TNF‐α), interleukin 1 beta (IL‐1β), and interleukin 6 (IL‐6) in BV2 cells with different treatments were measured according to the user guide of corresponding ELISA kits from Invitrogen. For quantification of Cox‐2, cell pellets were collected and the total protein was extracted with the reagents provided in Cox‐2 ELISA kit from Abcam, and the concentration of Cox‐2 was detected according to manual guide. For detection of Cox‐1, protein preparation and quantification of Cox‐1 in different treated groups were performed based on the guideline of Cox‐1 ELISA kit from MyBioSource. In signaling pathway study, cAMP was measured in BV2 cells after different treatments using Cyclic AMP Competitive ELISA Kit (Invitrogen).

### 4.3 Statistical analysis

All experiments involving imaging and quantification were repeated at least three times with similar results. For *in vivo* experiments, animal numbers in each group were indicated in figure legends. Data plotting and statistical tests were performed using GraphPad Prism software version 8.0. Data are represented as mean ± SEM and analyzed with unpaired two‐tailed Student's *t* test or one‐way ANOVA followed by Tukey's multiple comparisons test. In all graphs, statistical significance was described as ^*^p < 0.05, ^**^p < 0.01, ^***^p < 0.001, ^****^p < 0.0001; *^#^p* < 0.05, *^##^p* < 0.01, *^###^p* < 0.001, *^####^p* < 0.001; *^$$^p* < 0.01.

## CONFLICT OF INTEREST

The authors declare no competing interests.

## AUTHOR CONTRIBUTIONS

RL and BL designed the experiments. RL and YL performed experiments. RL and BL analyzed data and wrote the main manuscript text.

## Supporting information

Fig S1

Fig S2

Fig S3

Fig S4

Fig S5

Fig S6

Fig S7

Fig S8

Table S1

Table S2

Supplementary Material

## Data Availability

The data that support the findings of this study are available from the corresponding author upon reasonable request.

## References

[acel13566-bib-0001] Bach, C. T. , Creed, S. , Zhong, J. , Mahmassani, M. , Schevzov, G. , Stehn, J. , Cowell, L. N. , Naumanen, P. , Lappalainen, P. , Gunning, P. W. , & O'Neill, G. M. (2009). Tropomyosin isoform expression regulates the transition of adhesions to determine cell speed and direction. Molecular and Cellular Biology, 29(6), 1506–1514. 19124607 10.1128/MCB.00857-08 PMC2648248

[acel13566-bib-0002] Bishop, N. A. , Lu, T. , & Yankner, B. A. (2010). Neural mechanisms of ageing and cognitive decline. Nature, 464(7288), 529–535. 10.1038/nature08983 20336135 PMC2927852

[acel13566-bib-0003] Bogdan, D. , Falcone, J. , Kanjiya, M. P. , Park, S. H. , Carbonetti, G. , Studholme, K. , Gomez, M. , Lu, Y. , Elmes, M. W. , Smietalo, N. , Yan, S. , Ojima, I. , Puopolo, M. , & Kaczocha, M. (2018). Fatty acid‐binding protein 5 controls microsomal prostaglandin E synthase 1 (mPGES‐1) induction during inflammation. The Journal of Biological Chemistry, 293(14), 5295–5306. 10.1074/jbc.RA118.001593 29440395 PMC5892576

[acel13566-bib-0004] Campbell, M. , & Humphries, P. (2012). The blood‐retina barrier: Tight junctions and barrier modulation. Advances in Experimental Medicine and Biology, 763, 70–84. 23397619

[acel13566-bib-0005] Castaño, E. M. , Maarouf, C. L. , Wu, T. , Leal, M. C. , Whiteside, C. M. , Lue, L.‐F. , Kokjohn, T. A. , Sabbagh, M. N. , Beach, T. G. , & Roher, A. E. (2013). Alzheimer disease periventricular white matter lesions exhibit specific proteomic profile alterations. Neurochemistry International, 62(2), 145–156. 10.1016/j.neuint.2012.12.001 23231993 PMC3568229

[acel13566-bib-0006] Castellano, J. M. , Kirby, E. D. , & Wyss‐Coray, T. (2015). Blood‐borne revitalization of the aged brain. JAMA Neurology, 72(10), 1191–1194. 10.1001/jamaneurol.2015.1616 26237737 PMC4867550

[acel13566-bib-0007] Chen, M. , Luo, C. , Zhao, J. , Devarajan, G. , & Xu, H. (2019). Immune regulation in the aging retina. Progress in Retinal and Eye Research, 69, 159–172. 10.1016/j.preteyeres.2018.10.003 30352305 PMC6373845

[acel13566-bib-0008] Chevaleyre, V. , Heifets, B. D. , Kaeser, P. S. , Südhof, T. C. , & Castillo, P. E. (2007). Endocannabinoid‐mediated long‐term plasticity requires cAMP/PKA signaling and RIM1alpha. Neuron, 54(5), 801–812. 17553427 10.1016/j.neuron.2007.05.020 PMC2001295

[acel13566-bib-0009] Curthoys, N. M. , Freittag, H. , Connor, A. , Desouza, M. , Brettle, M. , Poljak, A. , Hall, A. , Hardeman, E. , Schevzov, G. , Gunning, P. W. , & Fath, T. (2014). Tropomyosins induce neuritogenesis and determine neurite branching patterns in B35 neuroblastoma cells. Molecular and Cellular Neurosciences, 58, 11–21. 24211701 10.1016/j.mcn.2013.10.011

[acel13566-bib-0010] Ghosh, M. , Aguirre, V. , Wai, K. , Felfly, H. , Dietrich, W. D. , & Pearse, D. D. (2015). The interplay between cyclic AMP, MAPK, and NF‐κB pathways in response to proinflammatory signals in microglia. BioMed Research International, 2015, 308461. 25722974 10.1155/2015/308461 PMC4334621

[acel13566-bib-0011] Häbig, K. , Gellhaar, S. , Heim, B. , Djuric, V. , Giesert, F. , Wurst, W. , Walter, C. , Hentrich, T. , Riess, O. , & Bonin, M. (1832). (2013) LRRK2 guides the actin cytoskeleton at growth cones together with ARHGEF7 and Tropomyosin 4. Biochimica Et Biophysica Acta (BBA) ‐ Molecular Basis of Disease, 12, 2352–2367. 10.1016/j.bbadis.2013.09.009 24075941

[acel13566-bib-0012] Haj‐Dahmane, S. , & Shen, R. Y. (2010). Regulation of plasticity of glutamate synapses by endocannabinoids and the cyclic‐AMP/protein kinase A pathway in midbrain dopamine neurons. The Journal of Physiology, 588(Pt 14), 2589–2604. 20498231 10.1113/jphysiol.2010.190066 PMC2916990

[acel13566-bib-0013] Haj‐Dahmane, S. , Shen, R. Y. , Elmes, M. W. , Studholme, K. , Kanjiya, M. P. , Bogdan, D. , Thanos, P. K. , Miyauchi, J. T. , Tsirka, S. E. , Deutsch, D. G. , & Kaczocha, M. (2018). Fatty‐acid‐binding protein 5 controls retrograde endocannabinoid signaling at central glutamate synapses. Proceedings of the National Academy of Sciences of the United States of America, 115(13), 3482–3487. 29531087 10.1073/pnas.1721339115 PMC5879704

[acel13566-bib-0014] Herkenham, M. , Lynn, A. B. , Johnson, M. R. , Melvin, L. S. , de Costa, B. R. , & Rice, K. C. (1991). Characterization and localization of cannabinoid receptors in rat brain: A quantitative in vitro autoradiographic study. The Journal of Neuroscience: the Official Journal of the Society for Neuroscience, 11(2), 563–583. 1992016 10.1523/JNEUROSCI.11-02-00563.1991 PMC6575215

[acel13566-bib-0015] Hill‐Burns, E. M. , Ross, O. A. , Wissemann, W. T. , Soto‐Ortolaza, A. I. , Zareparsi, S. , Siuda, J. , Lynch, T. , Wszolek, Z. K. , Silburn, P. A. , Mellick, G. D. , Ritz, B. , Scherzer, C. R. , Zabetian, C. P. , Factor, S. A. , Breheny, P. J. , & Payami, H. (2016). Identification of genetic modifiers of age‐at‐onset for familial Parkinson's disease. Human Molecular Genetics, 25(17), 3849–3862. 10.1093/hmg/ddw206 27402877 PMC5216611

[acel13566-bib-0016] Hui, X. , Li, H. , Zhou, Z. , Lam, K. S. L. , Xiao, Y. , Wu, D. , Ding, K. , Wang, Y. , Vanhoutte, P. M. , & Xu, A. (2010). Adipocyte fatty acid‐binding protein modulates inflammatory responses in macrophages through a positive feedback loop involving c‐Jun NH2‐terminal kinases and activator protein‐1. The Journal of Biological Chemistry, 285(14), 10273–10280. 10.1074/jbc.M109.097907 20145251 PMC2856232

[acel13566-bib-0017] Kamran, P. , Sereti, K. I. , Zhao, P. , Ali, S. R. , Weissman, I. L. , & Ardehali, R. (2013). Parabiosis in mice: A detailed protocol. Journal of Visualized Experiments: Jove, 6(80), 50556. 10.3791/50556 PMC3938334 24145664

[acel13566-bib-0018] Katsimpardi, L. , Litterman, N. K. , Schein, P. A. , Miller, C. M. , Loffredo, F. S. , Wojtkiewicz, G. R. , Chen, J. W. , Lee, R. T. , Wagers, A. J. , & Rubin, L. L. (2014). Vascular and neurogenic rejuvenation of the aging mouse brain by young systemic factors. Science, 344(6184), 630–634. 10.1126/science.1251141 24797482 PMC4123747

[acel13566-bib-0019] Kim, D. K. , & Han, D. , Park, J. , Choi, H. , Park, J. C. , Cha, M. Y. , Woo, J. , Byun, M. S. , Lee, D. Y. , Kim, Y. , & Mook‐Jung, I. (2019). Deep proteome profiling of the hippocampus in the 5XFAD mouse model reveals biological process alterations and a novel biomarker of Alzheimer's disease. Experimental & Molecular Medicine, 51(11), 1–17. 10.1038/s12276-019-0326-z PMC6856180 31727875

[acel13566-bib-0020] King, C. C. , Sastri, M. , Chang, P. , Pennypacker, J. , & Taylor, S. S. (2011). The rate of NF‐κB nuclear translocation is regulated by PKA and A kinase interacting protein 1. PLoS One, 6(4), e18713. 21556136 10.1371/journal.pone.0018713 PMC3083391

[acel13566-bib-0021] Li, Z. , Zeng, Y. , Chen, X. , Li, Q. , Wu, W. , Xue, L. , Xu, H. , & Yin, Z. Q. (2016). Neural stem cells transplanted to the subretinal space of rd1 mice delay retinal degeneration by suppressing microglia activation. Cytotherapy, 18(6), 771–784. 27067610 10.1016/j.jcyt.2016.03.001

[acel13566-bib-0022] Liets, L. C. , Eliasieh, K. , van der List, D. A. , & Chalupa, L. M. (2006). Dendrites of rod bipolar cells sprout in normal aging retina. Proceedings of the National Academy of Sciences of the United States of America, 103(32), 12156–12160. 10.1073/pnas.0605211103 16880381 PMC1524926

[acel13566-bib-0023] Liu, X. , Nemeth, D. P. , McKim, D. B. , Zhu, L. , DiSabato, D. J. , Berdysz, O. , Gorantla, G. , Oliver, B. , Witcher, K. G. , Wang, Y. , Negray, C. E. , Vegesna, R. S. , Sheridan, J. F. , Godbout, J. P. , Robson, M. J. , Blakely, R. D. , Popovich, P. G. , Bilbo, S. D. , & Quan, N. (2019). Cell‐type‐specific interleukin 1 receptor 1 signaling in the brain regulates distinct neuroimmune activities. Immunity, 50(2), 317–333.e316. 30683620 10.1016/j.immuni.2018.12.012 PMC6759085

[acel13566-bib-0024] Liu, Y. , Conboy, M. J. , Mehdipour, M. , & Liu, Y. (2017). Application of bio‐orthogonal proteome labeling to cell transplantation and heterochronic parabiosis. Nature Communications, 8(1), 643. 10.1038/s41467-017-00698-y PMC5608760 28935952

[acel13566-bib-0025] London, A. , Benhar, I. , & Schwartz, M. (2013). The retina as a window to the brain‐from eye research to CNS disorders. Nature Reviews Neurology, 9(1), 44–53. 10.1038/nrneurol.2012.227 23165340

[acel13566-bib-0026] Mansour, H. , Chamberlain, C. G. , Weible, M. W., II , Hughes, S. , Chu, Y. , & Chan‐Ling, T. (2008). Aging‐related changes in astrocytes in the rat retina: imbalance between cell proliferation and cell death reduces astrocyte availability. Aging Cell, 7(4), 526–540. 10.1111/j.1474-9726.2008.00402.x 18489730

[acel13566-bib-0027] Mattson, M. P. , & Magnus, T. (2006). Ageing and neuronal vulnerability. Nature Reviews Neuroscience, 7(4), 278–294. 10.1038/nrn1886 16552414 PMC3710114

[acel13566-bib-0028] Movsas, T. Z. , Wong, K. Y. , Ober, M. D. , Sigler, R. , Lei, Z. M. , & Muthusamy, A. (2018). Confirmation of Luteinizing Hormone (LH) in living human vitreous and the effect of LH receptor reduction on murine electroretinogram. Neuroscience, 385, 1–10. 29890291 10.1016/j.neuroscience.2018.05.049 PMC6063313

[acel13566-bib-0029] Oboudiyat, C. , Glazer, H. , Seifan, A. , Greer, C. , & Isaacson, R. S. (2013). Alzheimer's disease. Seminars in Neurology, 33(4), 313–329. 24234352 10.1055/s-0033-1359319

[acel13566-bib-0030] Oeckinghaus, A. , & Ghosh, S. (2009). The NF‐kappaB family of transcription factors and its regulation. Cold Spring Harbor Perspectives in Biology, 1(4), a000034. 20066092 10.1101/cshperspect.a000034 PMC2773619

[acel13566-bib-0031] Ozek, C. , Krolewski, R. C. , Buchanan, S. M. , & Rubin, L. L. (2018). Growth Differentiation Factor 11 treatment leads to neuronal and vascular improvements in the hippocampus of aged mice. Scientific Reports, 8(1), 17293. 10.1038/s41598-018-35716-6 30470794 PMC6251885

[acel13566-bib-0032] Poon, C. T. , Shah, K. , Lin, C. , Tse, R. , Kim, K. K. , Mooney, S. , Aubert, I. , Stefanovic, B. , & Hynynen, K. (2018). Time course of focused ultrasound effects on β‐amyloid plaque pathology in the TgCRND8 mouse model of Alzheimer's disease. Scientific Reports, 8(1), 14061. 30232364 10.1038/s41598-018-32250-3 PMC6145880

[acel13566-bib-0033] Ramírez, J. M. , Ramírez, A. I. , Salazar, J. J. , de Hoz, R. , & Triviño, A. (2001). Changes of astrocytes in retinal ageing and age‐related macular degeneration. Experimental Eye Research, 73(5), 601–615. 10.1006/exer.2001.1061 11747361

[acel13566-bib-0034] Runkle, E. A. , & Antonetti, D. A. (2011). The blood‐retinal barrier: Structure and functional significance. Methods in Molecular Biology (Clifton. N.J.), 686, 133–148. 10.1007/978-1-60761-938-3_5 21082369

[acel13566-bib-0035] Salvador, A. F. , de Lima, K. A. , & Kipnis, J. (2021) Neuromodulation by the immune system: A focus on cytokines. Nature Reviews. Immunology, 21(8), 526–541. 10.1038/s41577-021-00508-z 33649606

[acel13566-bib-0036] Samuel, M. A. , Voinescu, P. E. , Lilley, B. N. , de Cabo, R. , Foretz, M. , Viollet, B. , Pawlyk, B. , Sandberg, M. A. , Vavvas, D. G. , & Sanes, J. R. (2014). LKB1 and AMPK regulate synaptic remodeling in old age. Nature Neuroscience, 17(9), 1190–1197. 10.1038/nn.3772 25086610 PMC5369022

[acel13566-bib-0037] Samuel, M. A. , Zhang, Y. , Meister, M. , & Sanes, J. R. (2011). Age‐related alterations in neurons of the mouse retina. The Journal of Neuroscience: the Official Journal of the Society for Neuroscience, 31(44), 16033–16044. 10.1523/JNEUROSCI.3580-11.2011 22049445 PMC3238393

[acel13566-bib-0038] Satoh, A. , Imai, S. I. , & Guarente, L. (2017). The brain, sirtuins, and ageing. Nature Reviews. Neuroscience, 18(6), 362–374. 28515492 10.1038/nrn.2017.42

[acel13566-bib-0039] Senga, S. , Kobayashi, N. , Kawaguchi, K. , Ando, A. , & Fujii, H. (2018). Fatty acid‐binding protein 5 (FABP5) promotes lipolysis of lipid droplets, de novo fatty acid (FA) synthesis and activation of nuclear factor‐kappa B (NF‐κB) signaling in cancer cells. Biochimica Et Biophysica Acta. Molecular and Cell Biology of Lipids, 1863(9), 1057–1067. 29906613 10.1016/j.bbalip.2018.06.010

[acel13566-bib-0040] Smith, L. K. , He, Y. , Park, J.‐S. , Bieri, G. , Snethlage, C. E. , Lin, K. , Gontier, G. , Wabl, R. , Plambeck, K. E. , Udeochu, J. , Wheatley, E. G. , Bouchard, J. , Eggel, A. , Narasimha, R. , Grant, J. L. , Luo, J. , Wyss‐Coray, T. , & Villeda, S. A. (2015). β2‐microglobulin is a systemic pro‐aging factor that impairs cognitive function and neurogenesis. Nature Medicine, 21(8), 932–937. 10.1038/nm.3898 PMC4529371 26147761

[acel13566-bib-0041] Smith, L. K. , White, C. W., 3rd , & Villeda, S. A. (2018). The systemic environment: at the interface of aging and adult neurogenesis. Cell and Tissue Research, 371(1), 105–113. 29124393 10.1007/s00441-017-2715-8 PMC5748432

[acel13566-bib-0042] Soria Lopez, J. A. , González, H. M. , & Léger, G. C. (2019). Alzheimer's disease. Handbook of Clinical Neurology, 167, 231–255. 10.1055/s-0033-1359319 31753135

[acel13566-bib-0043] Telegina, D. V. , Kozhevnikova, O. S. , & Kolosova, N. G. (2018). Changes in retinal glial cells with age and during development of age‐related macular degeneration. Biochemistry Biokhimiia, 83(9), 1009–1017. 10.1134/S000629791809002X 30472939

[acel13566-bib-0044] Wyss‐Coray, T. (2016). Ageing, neurodegeneration and brain rejuvenation. Nature, 539(7628), 180–186. 10.1038/nature20411 27830812 PMC5172605

[acel13566-bib-0045] Yousef, H. , Czupalla, C. J. , Lee, D. , Chen, M. B. , Burke, A. N. , Zera, K. A. , Zandstra, J. , Berber, E. , Lehallier, B. , Mathur, V. , Nair, R. V. , Bonanno, L. N. , Yang, A. C. , Peterson, T. , Hadeiba, H. , Merkel, T. , Körbelin, J. , Schwaninger, M. , Buckwalter, M. S. , … Wyss‐Coray, T. , (2019). Aged blood impairs hippocampal neural precursor activity and activates microglia via brain endothelial cell VCAM1. Nature Medicine, 25(6), 988–1000. 10.1038/s41591-019-0440-4 PMC6642642 31086348

[acel13566-bib-0046] Yuan, T. F. , Liang, Y. X. , Peng, B. , Lin, B. , & So, K. F. (2015). Local proliferation is the main source of rod microglia after optic nerve transection. Scientific Reports, 5, 10788. 26035780 10.1038/srep10788 PMC4649910

[acel13566-bib-0047] Zhang, H. , Cherian, R. , & Jin, K. (2019). Systemic milieu and age‐related deterioration. GeroScience, 41(3), 275–284. 10.1007/s11357-019-00075-1 31152364 PMC6702503

