# Supplementary figures and images for: Accumulation of systematic TPM1 mediates inflammation and neuronal remodeling by phosphorylating PKA and regulating the FABP5/NF‐κB signaling pathway in the retina of aged mice
Source: Aging Cell. 2022 Feb 11;21(3):e13566. doi: 10.1111/acel.13566 (PMC8920455; doi:10.1111/acel.13566)

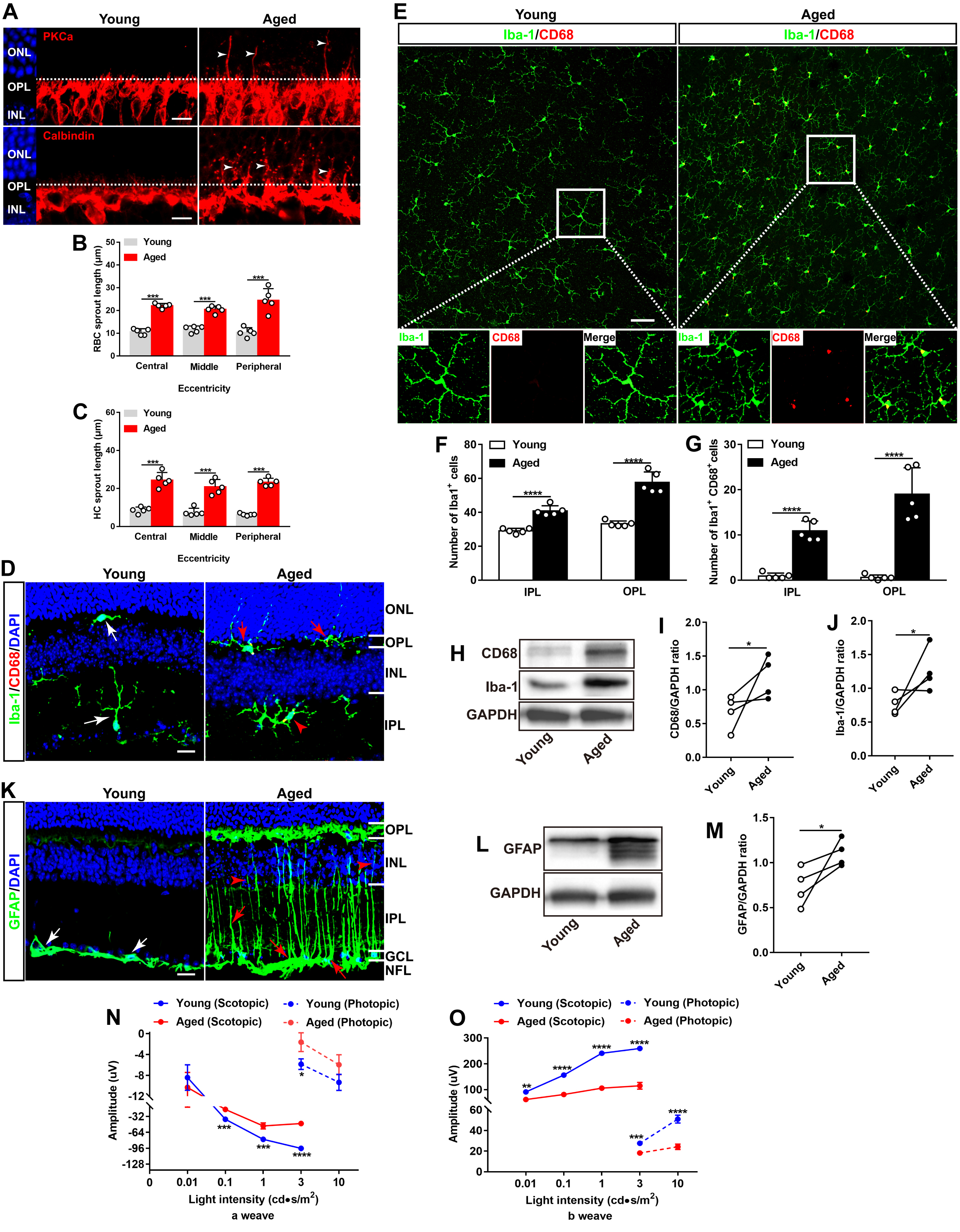

Supplement: Supplementary file 1 — Fig S1 [file ACEL-21-e13566-s001.jpg]

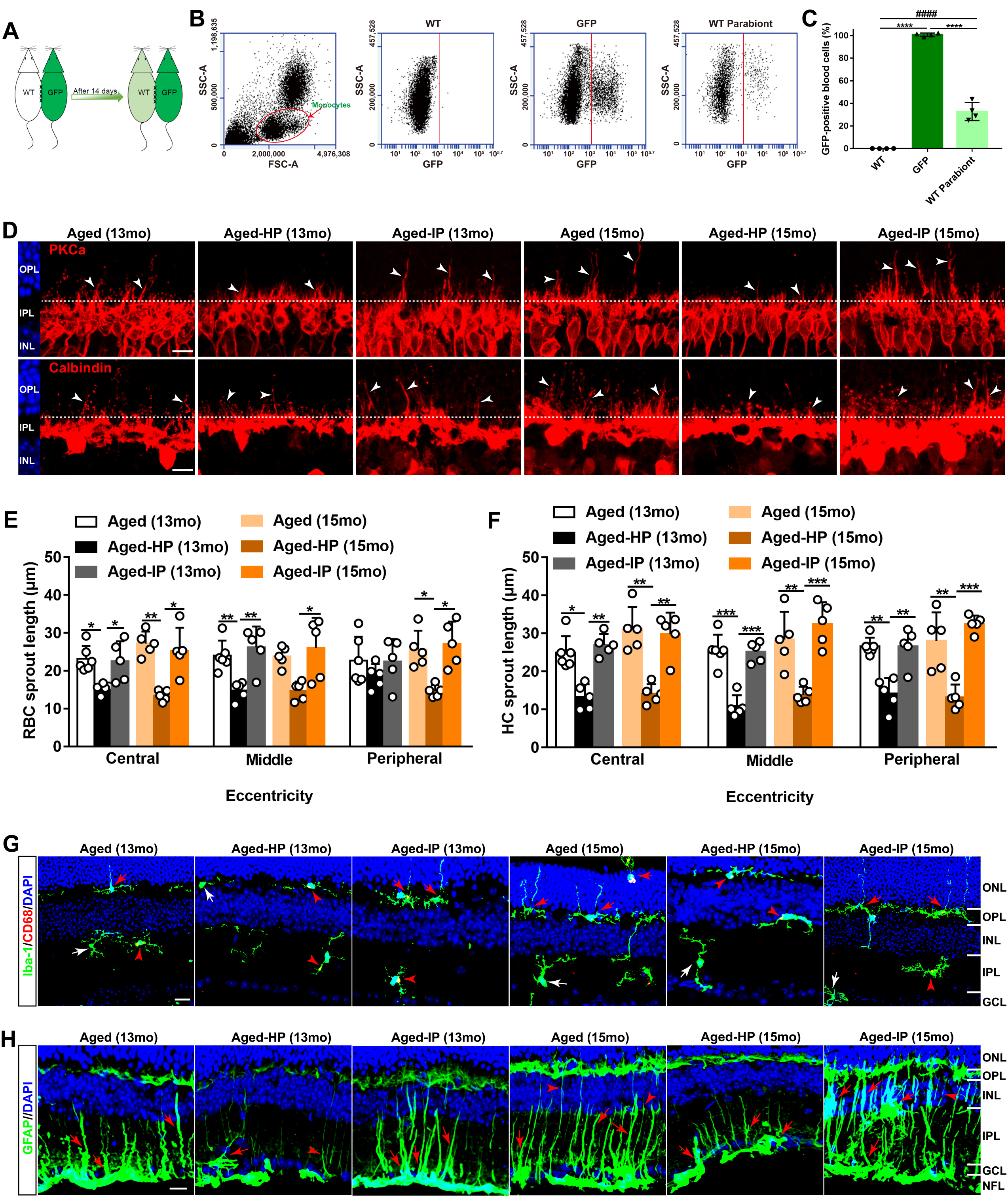

Supplement: Supplementary file 2 — Fig S2 [file ACEL-21-e13566-s004.jpg]

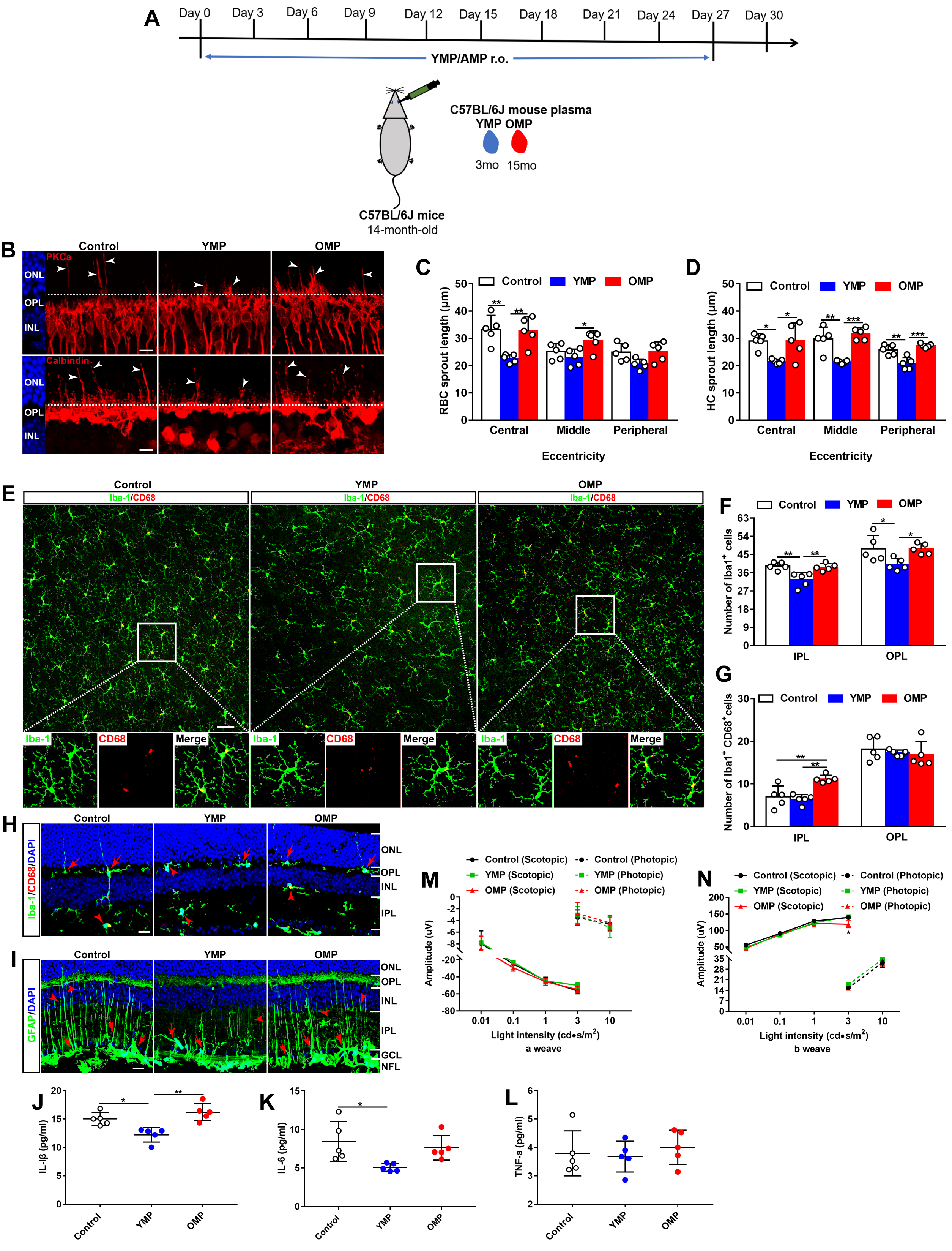

Supplement: Supplementary file 3 — Fig S3 [file ACEL-21-e13566-s002.jpg]

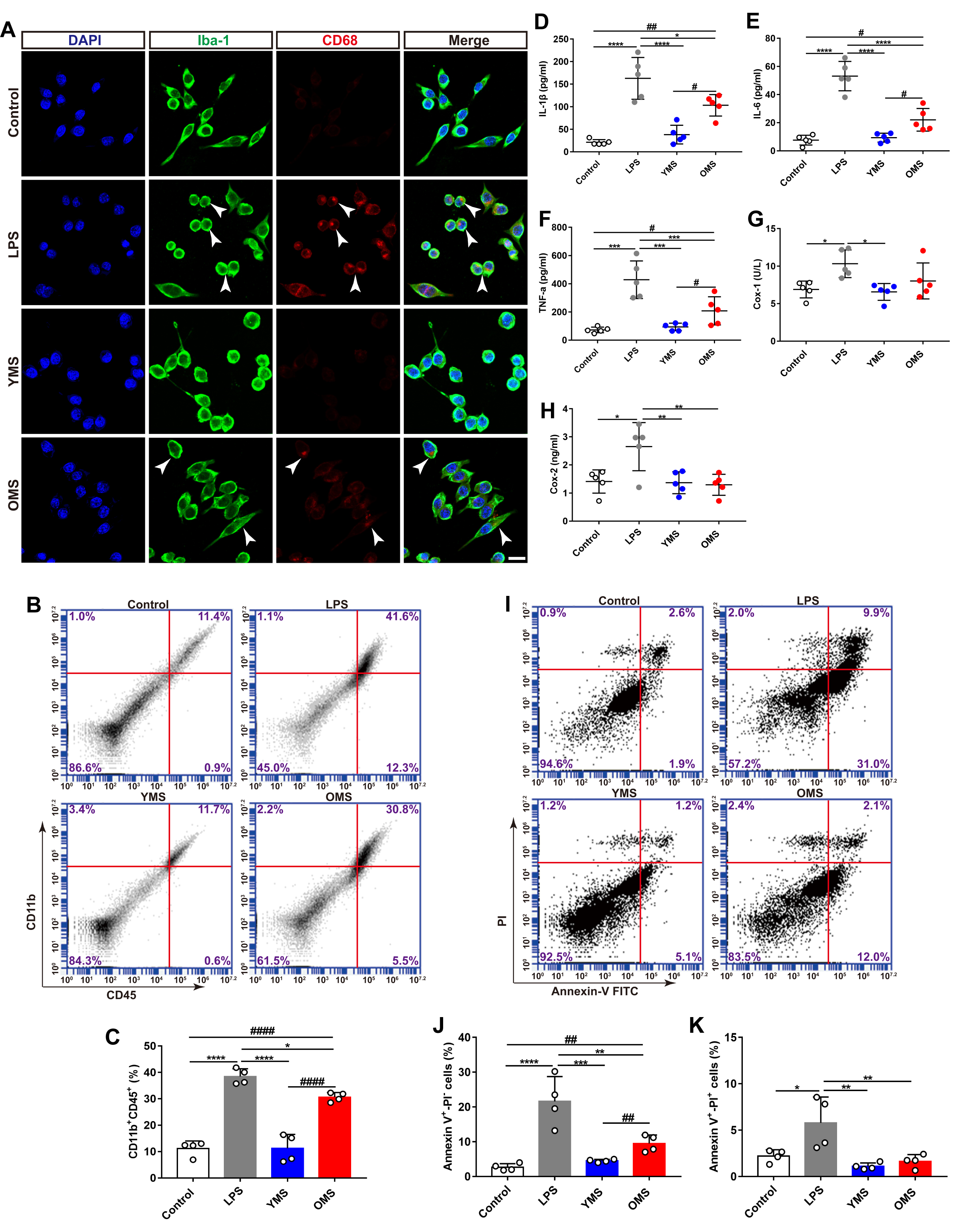

Supplement: Supplementary file 4 — Fig S4 [file ACEL-21-e13566-s008.jpg]

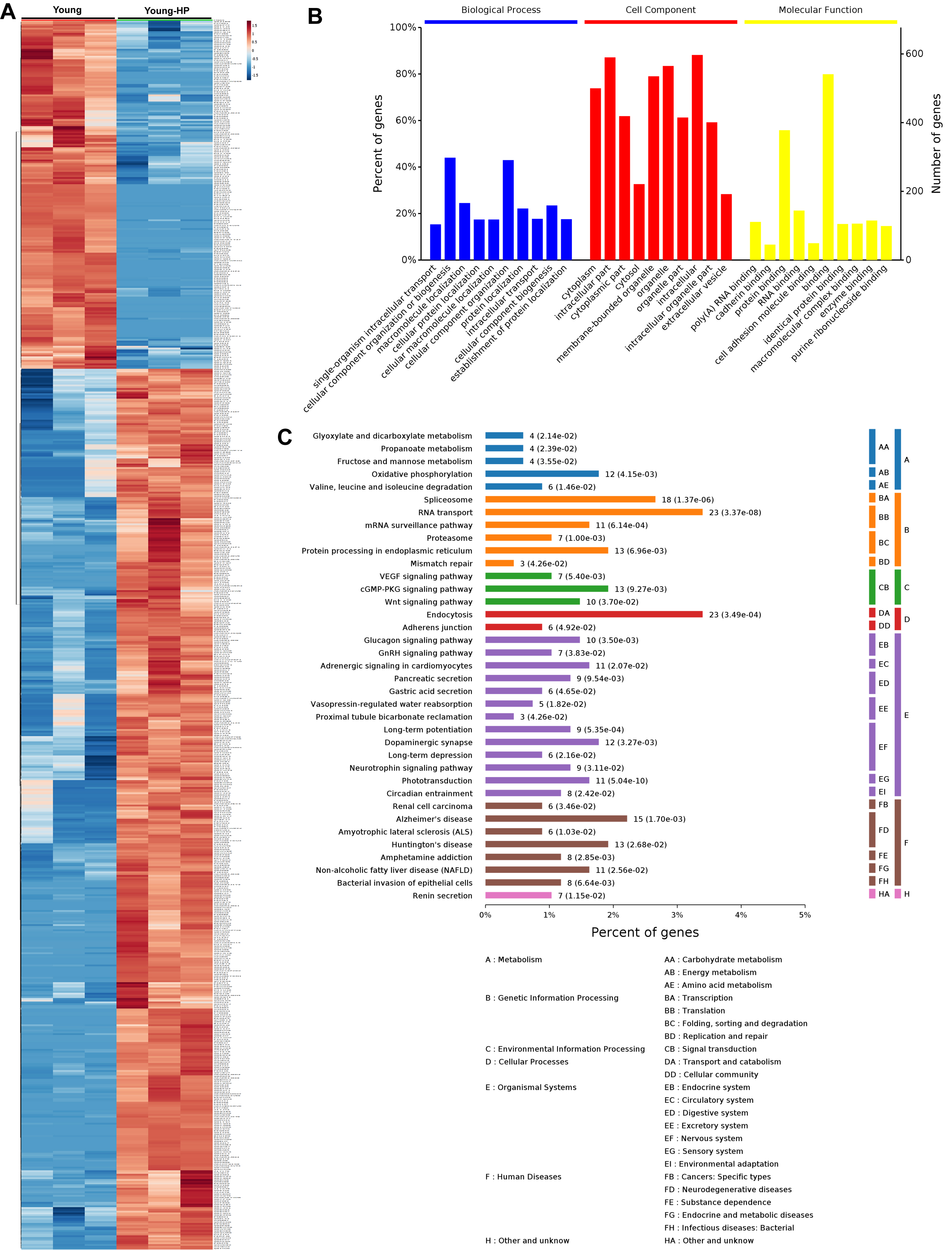

Supplement: Supplementary file 5 — Fig S5 [file ACEL-21-e13566-s009.jpg]

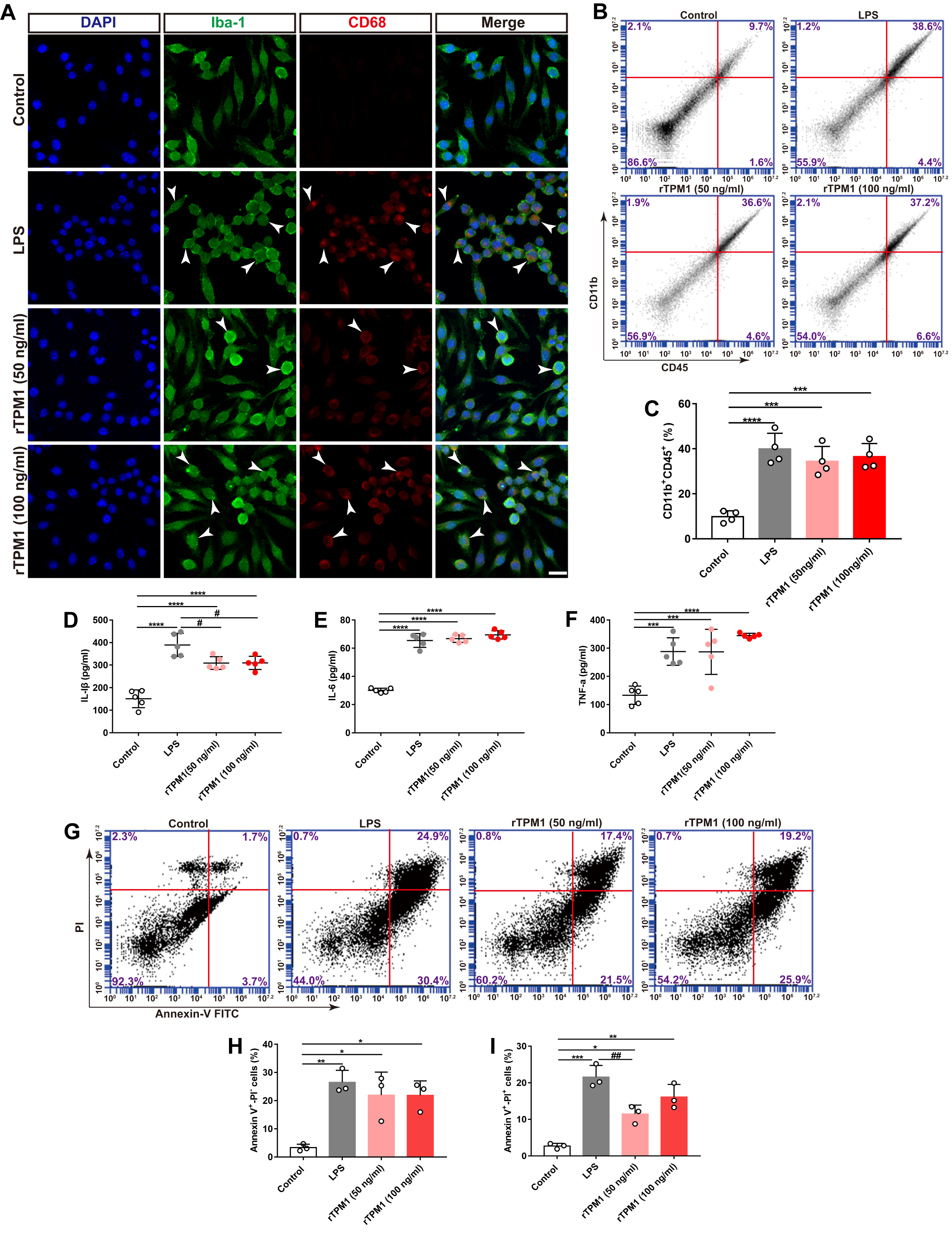

Supplement: Supplementary file 6 — Fig S6 [file ACEL-21-e13566-s007.jpg]

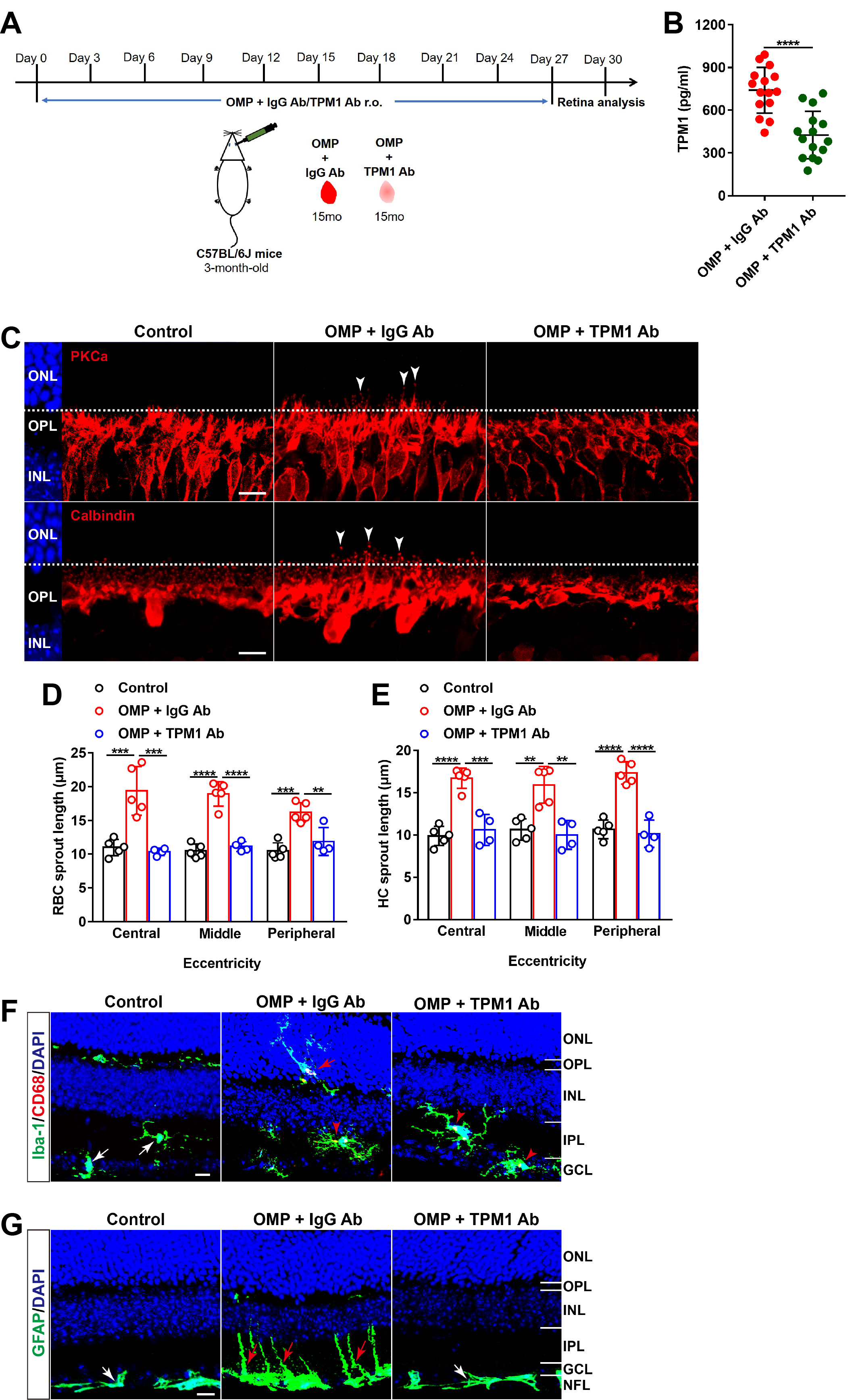

Supplement: Supplementary file 7 — Fig S7 [file ACEL-21-e13566-s010.jpg]

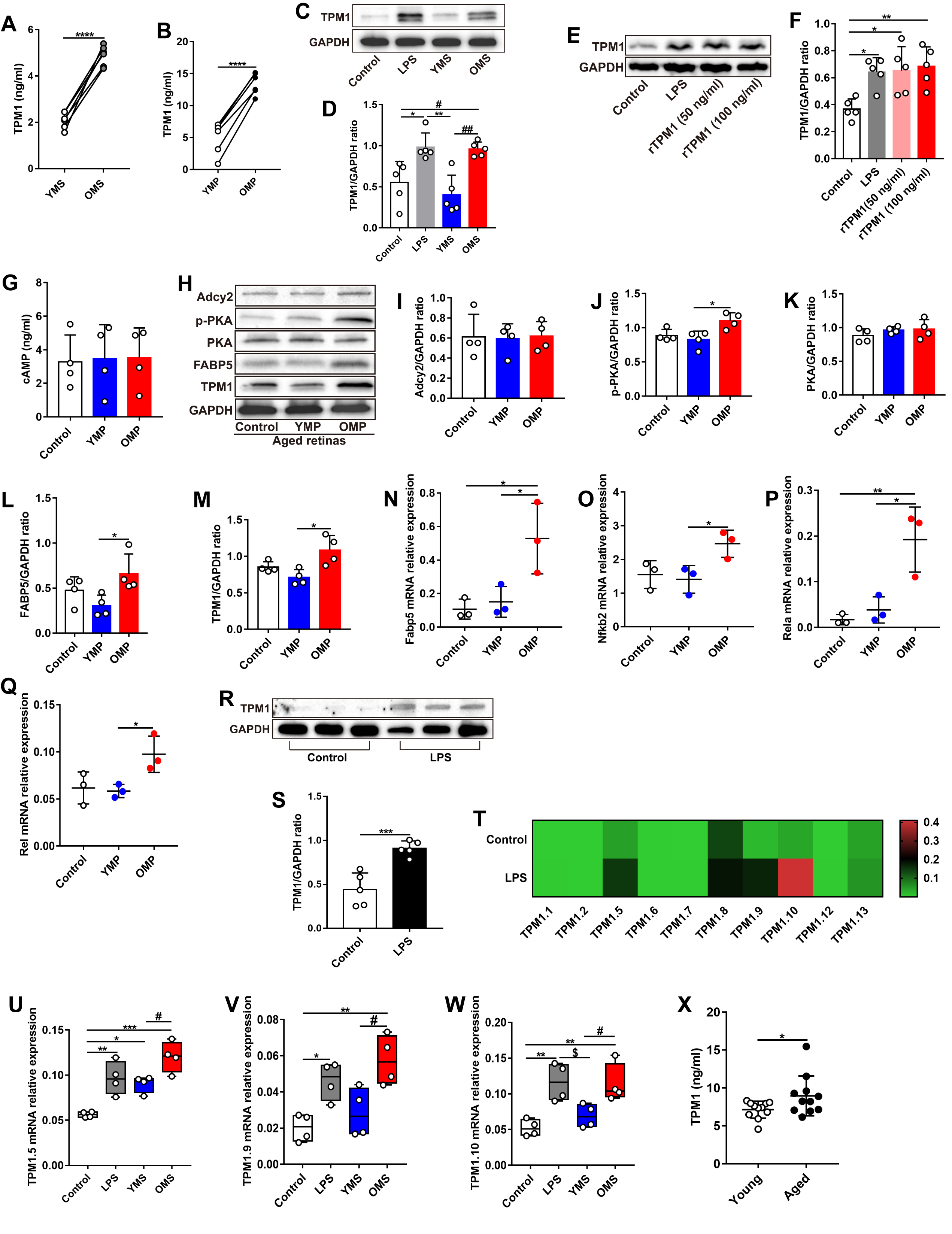

Supplement: Supplementary file 8 — Fig S8 [file ACEL-21-e13566-s006.jpg]
