# Supplementary material for: Accumulation of systematic TPM1 mediates inflammation and neuronal remodeling by phosphorylating PKA and regulating the FABP5/NF‐κB signaling pathway in the retina of aged mice
Source: Aging Cell. 2022 Feb 11;21(3):e13566. doi: 10.1111/acel.13566 (PMC8920455; doi:10.1111/acel.13566)
Supplement: Supplementary file 11 — Supplementary Material [file ACEL-21-e13566-s011.docx]

**Figure S1. Age-related alterations in neurons and glial cells in aged retinas. (A)** Representative confocal image of RBCs (top row) and HCs (bottom row) in retinal sections from young and aged mice. Scale bars, 10 µm. (**B-C)** Quantification of the aberrant sprout length of the dendrites of RBCs and HCs in young and aged retinas. Data are presented as mean ± SEM, n=5 mice in each group, unpaired two-tailed Student’s *t* test. **(D)** Retina sections from young and aged mice were stained with Iba-1and CD68. White arrow indicates CD68-negative microglia. Red arrowhead shows CD68-positive microglia and red arrows indicate the dendritic extension of activated microglia. Scale bars, 20 µm. (**E)** Double-staining of whole-mounted retinas from young and aged mice with Iba-1 and CD68. Representative confocal images focus on the OPL. The boxed regions are highly magnified at the bottom. Scale bars, 50 µm. (**F-G)** Quantification of the numbers of Iba-1^+^ and of CD68^+^Iba-1^+^ microglial cells in the IPL and OPL of whole-mounted young and aged retinas. Data are presented as mean ± SEM, n=5 mice in each group, unpaired two-tailed Student’s *t* test. (**H-J)** Western blot analysis (**H**) and quantification of CD68 and Iba-1 (**I-J**) in young and aged retinas. Data are presented as mean ± SEM, n=4 mice in each group, unpaired two-tailed Student’s *t* test. (**K)** Immunostaining of retinal sections from young and aged mice with GFAP antibody. White arrows show the resting astrocytes. Red arrows show activated astrocytes and red arrowheads indicate activated Müller cells. Scale bars, 20 µm. (**L-M)** Western blot analysis (**L**) and quantification of GFAP (**M**) in young and aged retinas. Data are presented as mean ± SEM, n=4 mice in each group, unpaired two-tailed Student’s *t* test. (**N-O)** ERG responses on young and aged mice. Data are presented as mean ± SEM, n=8 mice in each group, unpaired two-tailed Student’s *t* test.

**Figure S2 Heterochronic parabiosis mitigates age-related neuronal remodeling and inflammation in aged mouse retinas.** (**A)** Schematic diagram of the parabiosis model. Surgically joining of a WT mouse (C57BL/6J, white) to a Cx3cr1^+/GFP^ mouse (GFP mice, green). The presence of GFP-positive cells in the blood of the C57BL/6J partner was verified by flow cytometry 2 weeks after parabiosis. (**B-C)** Representative flow cytometry histograms showing the frequency of GFP^+^ cells in the blood of WT, GFP and WT parabiotic mice (**B**), and quantification of GFP^+^ cells (**C**). Data are presented as mean ± SEM and analyzed by one-way ANOVA with Tukey’s multiple comparison test, n=4 mice in each group. (**D)** Representative confocal images of RBCs (top row) and HCs (bottom row) in retinal sections from aged mice 2 and 4 months after parabiosis. Arrowheads indicate the aberrant dendritic sprouting of RBCs and HCs. Scale bars, 10 µm**. (E-F)** Quantification of the aberrant dendritic length of RBCs and HCs. Data are presented as mean ± SEM. n=6, 5, 5 in aged (13mo), aged-HP (13mo) and aged-IP (13mo), respectively; n=5, 5, 5 in aged (15mo), aged-HP (15mo) and aged-IP (15mo), respectively. One-way ANOVA analysis with Tukey’s multiple comparison test. (**G)** Retina sections from aged mice 2 and 4 months after parabiosis were stained with Iba-1and CD68. White arrows indicate CD68-negative microglia. Red arrowheads show CD68-positive microglia and red arrows indicate the dendritic extension of activated microglia. Scale bars, 20 µm. (**H)** Retina sections from aged mice 2 and 4 months after parabiosis were stained with GFAP antibody. Red arrows show activated astrocytes and red arrowheads indicate activated Müller cells. Scale bars, 20 µm.

**Figure S3 Young mouse plasma injection ameliorates age-related dendritic sprouting, inflammation and functional decline in aged mouse retinas.** (**A)** Schematic representation of the experimental design. Young mouse plasma (YMP) from three-month-old C57BL/6J mice or old mouse plasma (OMP) from fifteen-month-old C57BL/6J mice were retro-orbitally injected into aged C57BL/6J mice (14-month-old) for a total of ten times, 3 days apart, and retinas were collected at day 30 for further studies. **(B)** Representative confocal images of RBCs (top row) and HCs (bottom row) in retinal sections from aged mice after YMP or OMP treatment. Arrowheads indicate the aberrant dendritic sprouting of RBCs and HCs. Scale bars, 10 µm. (**C-D)** Quantification of the aberrant dendritic length of RBCs and HCs in aged retinas after YMP or OMP treatment. Data are presented as mean ± SEM. n=5 mice in each group. One-way ANOVA analysis with Tukey’s multiple comparison test. (**E)** Retina wholemounts from aged retinas after YMP or OMP treatment were stained with Iba-1 and CD68. Representative confocal images focus on the OPL. The boxed regions are highly magnified at the bottom showing the colocalization of CD68 with microglia. Scale bars, 50 µm. (**F-G)** Quantification of the numbers of Iba-1^+^ and of CD68^+^Iba-1^+^ microglia in the IPL and OPL of whole-mounted aged retina after YMP or OMP treatment. Data are presented as mean ± SEM. n=5 mice in each group. One-way ANOVA analysis with Tukey’s multiple comparison test. (**H)** Retina sections from aged mice after YMP or OMP treatment were stained with Iba-1 and CD68. Red arrowheads indicate CD68-positive microglia, and red arrows indicate the dendritic extension of activated microglia. Scale bars, 20 µm. (**I)** Retina sections from aged mice after YMP or OMP treatment were stained with GFAP antibody. Red arrows show activated astrocytes and red arrowheads indicate activated Müller cells. Scale bars, 20 µm. (**J-L)** ELISA analysis of IL-1β, IL-6 and TNF-α in aged retinas after YMP or OMP treatment. Data are presented as mean ± SEM. n=5 mice in each group. One-way ANOVA analysis with Tukey’s multiple comparison test. (**M-N)** ERG recordings on aged mice after YMP or OMP treatment. Data are presented as mean ± SEM. n=12 mice in each group. One-way ANOVA with Tukey’s multiple comparison test.

**Figure S4 Old mouse serum treatment induces neuroinflammation and apoptosis in BV2 cells.** (**A)** Immunostaining of BV2 cells with Iba-1 and CD68 after treatment with LPS, YMS or OMS. Arrowheads show colocalization of microglial cells with CD68. Scale bar, 20 µm. (**B)** Flow cytometry analysis of activated microglia stained with FITC-conjugated anti-CD45 and phycoerythrin (PE)-conjugated anti-CD11b antibodies after treatment with LPS, YMS or OMS. Activated microglia (CD11b^+^CD45^+^) were gated. (**C)** Quantification of CD11b^+^CD45^+^ cells. Data are presented as mean ± SEM and analyzed by one-way ANOVA with Tukey’s multiple comparison test (compared to LPS, *^*^p*<0.05, *^****^p*<0.001; compared to OMS, *^####^p*<0.001). Four independent experiments were performed. (**D-H)** ELISA analysis of IL-1β, IL-6, TNF-α, cyclooxygenase (COX)-1 and COX-2 in BV2 cells after LPS, YMS or OMS treatment. Data are presented as mean ± SEM and analyzed by one-way ANOVA with Tukey’s multiple comparison test (compared to LPS, *^*^p*<0.05 *^**^p*<0.01, *^***^p*<0.001, *^****^p*<0.001; compared to OMS, *^#^p*<0.05, *^##^p*<0.01). Five independent experiments were performed. (**I)** Flow cytometry analysis of cell apoptosis in BV2 cells after treatment with LPS, YMS or OMS. Cells were stained with Annexin-V and propidium iodide (PI). Percentages of early apoptosis cells (annexin-V^+^PI^-^) and late apoptosis cells (annexin-V^+^PI^+^) within each quadrant are indicated. (**J-K)** Quantification of annexin-V^+^PI^-^ and of annexin-V^+^PI^+^ cells. Data are presented as mean ± SEM and analyzed by one-way ANOVA with Tukey’s multiple comparison test (compared to LPS, *^*^p*<0.05 *^**^p*<0.01, *^***^p*<0.001, *^****^p*<0.001; compared to OMS, *^##^p*<0.01), four independent experiments were performed.

**Figure S5 Analysis of differentially expressed proteins by LC-MS/MS.** (**A)** A heat map showing the up- or down-regulation of 699 differentially expressed proteins (DEPs) between young unpaired (3mo) and young-HP (5mo) groups (n=3 mice/group). (**B)** Gene ontology (GO) enrichment analysis of DEPs between young unpaired (3mo) and young-HP (5mo) mice retinas. Top-ten significantly enriched GO terms corresponding to biological process (blue color), cellular component (red color) and molecular function (yellow color) were presented. The Y-axes indicate the percentage (the left axis) and number (the right axis) of DEPs. (**C)** KEGG pathway enrichment analysis of DEPs between young unpaired (3mo) and young-HP (5mo) mice retinas. The numbers of DEPs in a specific pathway and corresponding p-values are shown next to each specific bar.

**Figure S6 Treatment by recombinant TPM1 protein induces neuroinflammation and cell apoptosis in BV2 cells.** (**A)** BV2 cells were stained with Iba-1 and CD68 after LPS or rTPM1 protein treatment. Arrowheads show colocalization of microglial cells with CD68. Four independent experiments were performed with two repeats. Scale bar, 20 µm. (**B)** Flow cytometry analysis of activated microglia in BV2 cells after LPS or rTPM1 protein treatment. Cells were stained with FITC-conjugated anti-CD45 and phycoerythrin (PE)-conjugated anti-CD11b antibodies and activated microglia (CD11b^+^CD45^+^) were gated. (**C)** Quantification of CD11b^+^CD45^+^ cells. Data are presented as mean ± SEM and analyzed by one-way ANOVA with Tukey’s multiple comparison test. Four independent experiments were performed. (**D-F)** ELISA analysis of cytokine IL-1β, IL-6 and TNF-α in BV2 cells after LPS or rTPM1 protein treatment. Data are presented as mean ± SEM and analyzed by one-way ANOVA with Tukey’s multiple comparison test (compared to Control, *^***^p*<0.001 *^****^p*<0.001; Compared to LPS, *^#^p*<0.05). Four independent experiments were performed. (**G)** Flow cytometry analysis of cell apoptosis in BV2 cells after exposure to LPS or rTPM1 protein. Cells were stained with Annexin-V and PI. Percentages of early apoptosis cells (annexin-V^+^PI^-^) and late apoptosis cells (annexin-V^+^PI^+^) within each quadrant are indicated. (**H-I)** Quantification of annexin-V^+^PI^-^ and of annexin-V^+^PI^+^ cells. Data are presented as mean ± SEM and analyzed by one-way ANOVA with Tukey’s multiple comparison test (compared to Control, *^*^p*<0.05, *^**^p*<0.01, *^***^p*<0.001, Compared to LPS, *^##^p*<0.01), three independent experiments were performed.

**Figure S7 Old mouse plasma with TPM1 depletion failed to induce age-related phenotype in young mice.** (**A**) Schematic representation of the experimental design. Old mouse plasma (OMP) with TPM1 depletion (OMP + TPM1 Ab) or IgG control (OMP + IgG Ab) were retro-orbitally injected into young C57BL/6J mice (3-month-old) for a total of ten times, 3 days apart, and retinas were collected at day 30 for further studies. (**B**) ELISA analysis of TPM1 in OMP after immunoprecipitation. Data are presented as mean ± SEM (n=15 mice in each group; Unpaired two-tailed Student’s t test). (**C**) Representative confocal images of RBCs (top row) and HCs (bottom row) in retinal sections from young mice after TPM1-depleted OMP or OMP control treatment. Arrowheads indicate the aberrant dendritic sprouting of RBCs and HCs. Scale bars, 10 µm. (**D-E**) Quantification of the aberrant dendritic length of RBCs and HCs in aged retinas after TPM1-depleted OMP or OMP control treatment. Data are presented as mean ± SEM (n=5 mice in each group. One-way ANOVA analysis with Tukey’s multiple comparison test). (**F**) Retina sections from young mice after TPM1-depleted OMP or OMP control treatment were stained with Iba-1 and CD68. Red arrowheads indicate CD68-positive microglia, and red arrow indicates the dendritic extension of activated microglia. White arrows indicate the resting microglia. Scale bars, 20 µm. (**G**) Retina sections were stained with GFAP antibody. Red arrows show activated astrocytes and white arrows show the resting astrocytes. Scale bars, 20 µm.

**Figure S8 Systematic TPM1 exerts its function by phosphorylating PKA and activating FABP5/NF-κB signaling pathway.** (**A-B)** ELISA analysis of TPM1 in serum (**A**) and plasma (**B**) of young or aged mice. Data are presented as mean ± SEM and analyzed with unpaired two-tailed Student’s *t* test. (YMP vs. OMP, *^****^p*<0.001, YMS vs. OMS, *^****^p*<0.001). Six independent experiments were performed. (**C-D)** Western blot analysis (**C**) and quantification of TPM1 (**D**) in BV2 cells after LPS, YMS or OMS treatment. Data are presented as mean ± SEM and analyzed by one-way ANOVA with Tukey’s multiple comparison test (Compared to LPS, *^*^p*<0.05, *^**^p*<0.01; compared to OMS, *^#^p*<0.05, *^##^p*<0.01). Five independent experiments were performed. (**E-F)** Western blot analysis (**E**) and quantification of TPM1 (**F**) in BV2 cells after LPS or rTPM1 protein treatment. Data are presented as mean ± SEM and analyzed by one-way ANOVA with Tukey’s multiple comparison test (compared to Control, *^*^p*<0.05, *^**^p*<0.01). Five independent experiments were performed. (**G)** ELISA analysis of cAMP in aged retinas after YMP or OMP treatment. Data are presented as mean ± SEM. n=4 mice in each group. One-way ANOVA analysis with Tukey’s multiple comparison test. (**H-M)** Western blot analysis (**H**) and quantification of Adcy2, p-PKA, PKA, FABP5 and TPM1 (**I-M**) in aged retinas after YMP or OMP treatment. Data are presented as mean ± SEM. n=4 mice in each group. One-way ANOVA analysis with Tukey’s multiple comparison test. (**N-Q)** qPCR analysis of Fabp5, Nfκb2, Rela and Rel in aged retinas after YMP or OMP treatment. Data are presented as mean ± SEM. n=3 mice in each group. One-way ANOVA analysis with Tukey’s multiple comparison test. (**R-S)** Western blot analysis (**R**) and quantification of TPM1 (**S**) in BV2 cells after LPS treatment. Data are presented as mean ± SEM and analyzed with unpaired two-tailed Student’s *t* test. Five independent experiments were performed. (**T)** A heat map showing the mRNA expression levels of TPM1 isoforms in BV2 cells after LPS treatment. The mRNA levels of TPM1 isoforms (TPM1.1, TPM1.2, TPM1.5, TPM1.6, TPM1.7, TPM1.8, TPM1.9, TPM1.10, TPM1.12 and TPM1.13) were quantified by qRT-PCR. Five independent experiments were performed in triplicate. (**U-W)** qPCR analysis of TPM1.5, TPM1.9 and TPM1.10 in BV2 cells after LPS, YMS or OMS treatment. Four independent experiments were performed. Data are presented as mean ± SEM and analyzed by one-way ANOVA with Tukey’s multiple comparison test (compared to Control, *^*^p*<0.05, *^**^p*<0.01, *^***^p*<0.001; Compared to OMS, *^#^p*<0.05; Compared to LPS, *^$^p*<0.05). Four independent experiments were performed. (**X**) ELISA analysis of TPM1 in human blood plasma from both young and aged donors. Data are presented as mean ± SEM and analyzed with unpaired two-tailed Student’s *t* test. (Young vs. Aged, *^*^p*<0.05).
